# An apicoplast-localized GTPase is essential for *Toxoplasma gondii* survival

**DOI:** 10.1128/msphere.00713-25

**Published:** 2025-12-09

**Authors:** Michael B. Griffith, Morgan E. Wagner, Victoria L. Robinson, Aoife T. Heaslip

**Affiliations:** 1 Department of Molecular and Cell Biology, University of Connecticut 124501 , Storrs, Connecticut, USA; Virginia-Maryland College of Veterinary Medicine, Blacksburg, Virginia, USA

**Keywords:** *Toxoplasma gondii*, apicoplast, GTPase

## Abstract

The apicoplast is an essential organelle found in Apicomplexa, a large phylum of intracellular eukaryotic pathogens. The apicoplast produces metabolites that are utilized for membrane biogenesis and energy production. A majority of apicoplast-resident proteins are encoded by the nuclear genome and are trafficked to the apicoplast and are referred to as nuclear-encoded and apicoplast-trafficked (NEAT) proteins. In this study, we characterized a NEAT protein named TgBipA, which is a homolog of the highly conserved prokaryotic translational GTPase BipA. BipA is essential for bacterial survival in stress conditions and functions through interactions with the prokaryotic ribosome, although its role is not fully understood. Through genetic knockouts of TgBipA and immunofluorescence imaging, we show that the loss of TgBipA results in apicoplast genome replication defects, disruption of NEAT trafficking, loss of the apicoplast, and ultimately parasite death. Furthermore, we show through comparative studies that this phenotype closely resembles the delayed death phenomenon observed when inhibiting apicoplast translation. Finally, we show that TgBipA is an active GTPase *in vitro,* and its GTP hydrolysis activity is critical for its cellular function. Our findings demonstrate that TgBipA is a GTPase that has an essential role in apicoplast maintenance, providing new insights into the cellular processes of the organelle.

**IMPORTANCE:**

*Toxoplasma gondii,* and many other parasites in the phylum Apicomplexa, are pathogens with significant medical and veterinary importance. Most Apicomplexa contain a non-photosynthetic plastid organelle named the apicoplast. This organelle produces essential metabolites, and perturbation of apicoplast function results in parasite death. The apicoplast contains bacterial-like pathways for apicoplast genome replication and expression. Thus, the discovery of the apicoplast leads to optimism that this organelle would provide a wealth of anti-parasitic drug targets. Therefore, the identification and characterization of new apicoplast proteins could provide new opportunities for therapeutic development. In this study, we characterized the function of a protein called TgBipA, a homolog of a highly conserved bacterial GTPase BipA, which has been implicated in the maturation of the 50S ribosomal subunit and adaptation to cellular stress. We show that TgBipA is essential for apicoplast maintenance and parasite survival.

## INTRODUCTION

The Apicomplexan phylum is a diverse group of obligate intracellular parasites, including *Toxoplasma gondii*, *Plasmodium* spp., and *Babesia* spp.; the causative agents of human diseases toxoplasmosis, malaria, and babesiosis, respectively (1–4). *T. gondii* can cause life-threatening disease when infection occurs *in utero* or in the immunocompromised (1, 2, 5, 6). For a successful infection, the parasite must survive within cells of its host, where it relies on nutrients acquired from the host cell and metabolites formed *de novo* (7–9). *T. gondii*, along with many other Apicomplexans, relies on a non-photosynthetic plastid organelle, termed the apicoplast, to produce essential metabolites (9). The apicoplast is a four-membraned organelle derived from secondary endosymbiosis of a red algae, and thus is prokaryotic in origin (9). It houses four essential metabolic pathways: a fatty acid type II (FASII) (10), heme precursor (11), isopentenyl pyrophosphate (12), and lysophosphatidic acid pathways (13). In addition, iron-sulfur cluster biosynthesis and ferredoxin redox pathways provide cofactors for metabolic enzymes (12, 14–16).

Most apicoplast proteins are nuclear-encoded, the so-called nuclear-encoded apicoplast-trafficked (NEAT) pool of proteins (17–19). Thus, organelle function requires trafficking these proteins to the apicoplast. Many NEAT proteins contain an N-terminal bipartite signaling motif that consists of a signal peptide (SP) that directs the protein to the ER during translation, and an apicoplast targeting motif. Removal of the SP in the ER reveals an apicoplast targeting sequence (17, 18, 20). The apicoplast transit peptide can vary greatly in length and sequence but tends to have an overall positive charge (17, 20–22). This variation has led to difficulties in identifying putative transit peptides through the use of prediction algorithms (23, 24). Other NEAT proteins contain neither an SP nor an identifiable transit peptide. What sequences guide these proteins to the ER, and subsequently the apicoplast, is unclear (21, 25–29). Membrane-associated, transmembrane (TM) domain containing NEAT proteins traffic to the apicoplast through ER-derived vesicular transport that occurs in a cell-cycle-dependent manner (30, 31). How lumenal proteins are trafficked is poorly understood, as these proteins are not observed in trafficking vesicles in wild-type (WT) parasites. Once NEAT lumen proteins have traversed the outermost apicoplast membrane, proteins are then trafficked across the three innermost membranes using a modified ERAD complex and TIC and TOC protein translocons (28, 29, 32). Translocation into the lumen is followed by cleavage of the transit peptide to form the fully mature protein (33, 34).

In addition to the nuclear-encoded proteome, the apicoplast contains its own 35 Kb genome (35) encoding tRNAs, some 70S ribosomal components, RNA polymerase components, and two genes not directly involved in translation; *SufB* part of the an iron-sulfur cluster pathway (14) and *ClpC* which is speculated to be involved in protein translocation into the apicoplast lumen (35–37). Despite the low number of metabolic proteins translated from the apicoplast genome, several antibiotics that target bacterial translation also have anti-parasitic effects in *Toxoplasma* and other Apicomplexa and are in clinical use, highlighting the importance of apicoplast translation (38–42). Unfortunately, the utility of apicoplast translation targeting drugs has been somewhat limited by the so-called delayed death effect, whereby there are no apparent growth defects in the first intracellular growth cycle, but parasite death begins in the second intracellular cycle (42–45). Although the mechanism of action of these antibiotics is well characterized in bacteria, it is poorly understood in parasites.

Apicoplast proteins are attractive targets for anti-parasitic drug development, due to their similarity to prokaryotic, rather than eukaryotic, proteins. Given the importance of apicoplast translation for parasite viability and therapeutics, we sought to identify proteins with roles in this process. Our laboratory had previously characterized a bacterial GTPase, BipA (hereafter referred to as bBipA), which had been implicated in 50S ribosomal maturation as well as other adaptive processes associated with stress (46–52). *T. gondii* contains a homolog of this protein (TgME49_301380) that is 65% similar to its bacterial counterpart. The predicted apicoplast localization (53) and essentiality of this protein (54) motivated us to study its function in the parasite.

We created an inducible TgBipA knockout parasite line and showed that loss of TgBipA results in a cascade of phenotypic effects starting with a disruption to apicoplast genome maintenance. Next, vesicles containing NEAT proteins accumulate adjacent to the apicoplast. This coincides with a disruption to apicoplast morphology and eventual loss of the organelle. Similar phenotypic effects were observed when apicoplast translation was inhibited by doxycycline (DOX), but the timing of these events varied between the two conditions. While parasite death begins ~72 h after translation inhibition or loss of TgBipA, we were surprised to observe parasites that survived for up to 9 days after treatment, demonstrating that parasite death does not occur strictly in the second lytic cycle, as previously reported for the delayed death effect (37, 43, 48, 49, 55). Finally, we demonstrate that TgBipA is an active GTPase and that its ability to hydrolyze GTP is critical for its function. Collectively, these results show that TgBipA plays an essential role in apicoplast maintenance.

## RESULTS

### TgME49_301380 is an apicoplast-localized homolog of bBipA

**Fig 1:**
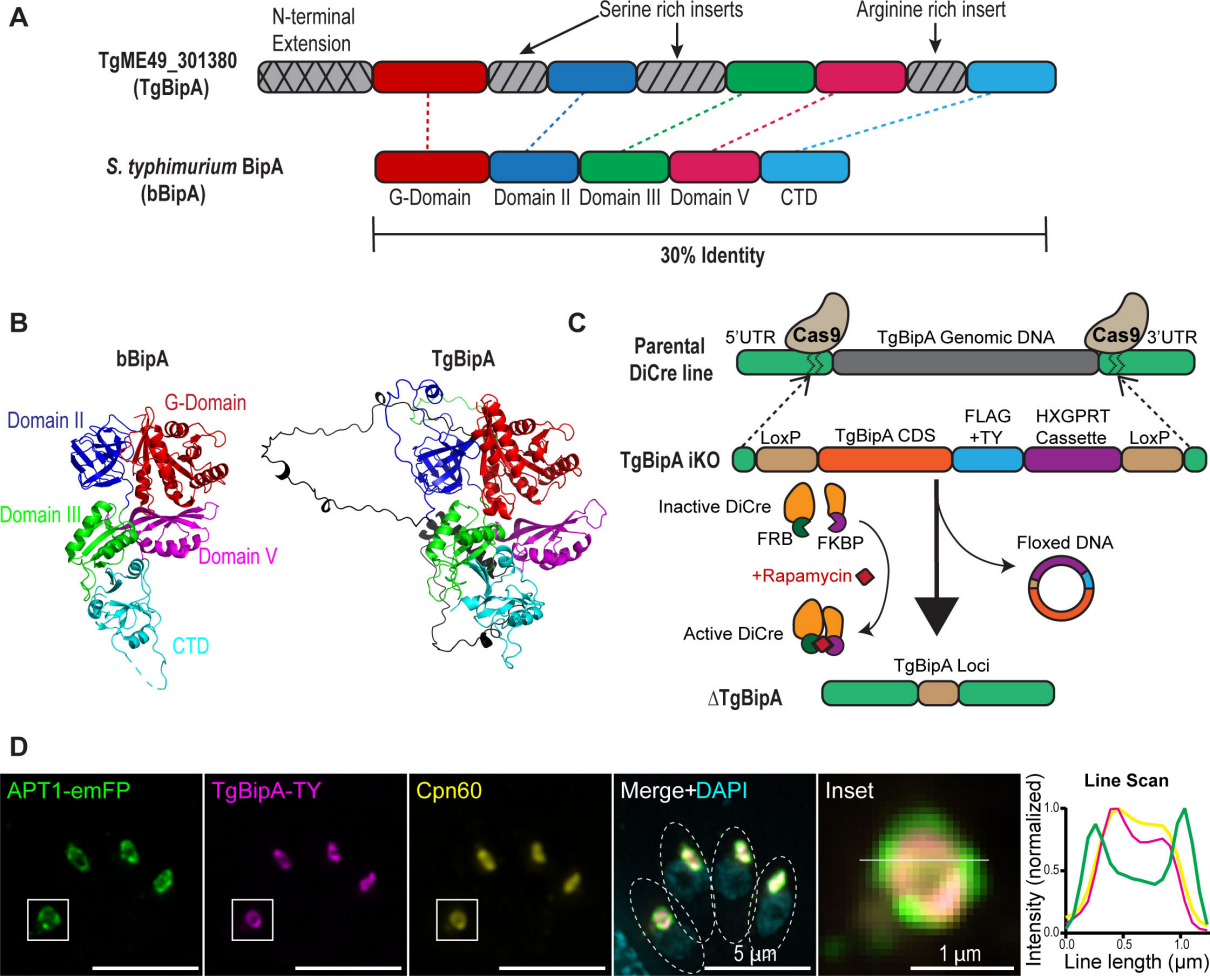
TgBipA is an apicoplast-localized protein and homolog of BipA. (**A**) Domain conservation between TgBipA and *S. typhimurium* BipA. TgBipA contains an N-terminal extension and three inserts not found in BipA. (**B**) Comparison of *S. typhimurium* BipA protein structure (PDB 8EWH) and AF 3 model prediction of TgBipA, minus the first 500 amino acids corresponding to the N-terminal extension. TgBipA unique inserts are in black. (**C**) Schematic of the TgBipA genomic locus in parental, TgBipA-iKO, and TgBipA knockout (ΔTgBipA) parasites. In the RH_Ku80:Dicre:T2A:CAT parasite line, the endogenous TgBipA gene was replaced with LoxP-flanked TgBipA coding sequence containing a FLAG and TY tag, followed by an HXGPRT drug selection cassette. Rapamycin treatment results in the activation of Cre recombinase and results in the excision of the floxed construct, resulting in TgBipA deletion. (**D**) Immunofluorescence showing colocalization between TgBipA using anti-Ty antibody (magenta) and the apicoplast membrane marker APT1 fused to emFP (green), and the apicoplast lumen marker Cpn60 labeled using an anti-Cpn60 antibody (yellow). Merge + DAPI (cyan) shows a juxtanuclear position of the apicoplast. Dotted circles denote parasite outlines. Inset of apicoplast with line drawn indicating the region used to make line intensity profile, which shows APT1 surrounding TgBipA and Cpn60 signals. Images are maximum intensity projections of deconvolved images. Scale bar = 5 µm. Inset scale bar = 1 µm.

To define the biochemical and structural features of TgBipA, we performed sequence alignments between *Salmonella typhimurium* bBipA and TgME49_301380 (Fig. 1A; Fig. S1) (50). TgBipA differs from bBipA in that it contains three insertions, two are serine rich (residues 215–256 and 308–394), and the other is arginine rich (residues 642–714). It also has a ~520 amino acid N-terminal extension that likely contains the signal motifs required for apicoplast trafficking (17), although the protein does not contain an identifiable SP based on primary sequence. TgBipA (amino acids 521–1,333) shares 41% identity with bBipA (51). The four conserved G-motifs are readily recognizable within the GTPase (G-) domain of TgBipA, which shares 55% identity with the G-domain of bBipA (52). To gain insight into the structural features of the protein, we modeled TgBipA using Alphafold (AF) (Fig. 1B) (56). The predicted structure suggests that the core of the protein, defined by five domains including a G-domain, OB fold, two alpha-beta domains, and a C-terminal domain unique to the BipA family, is similar in both proteins. The pLDDT scores, which denote the confidence of the prediction, are high throughout this part of the model (>90). In contrast, AF was unable to define any structural features in the three large insertions (pLDDT <50). This low confidence score suggested they are intrinsically disordered (IDR), subsequently confirmed by a PONDR-FIT (57) assessment of the protein (Fig. S1).

To study the role of TgBipA, we created a TgBipA inducible knockout line, henceforth referred to as TgBipA-iKO, using the previously established DiCre system (58). This line expresses Cre recombinase split into two components that are tagged with the rapamycin (rapa)-binding peptides (FRB and FBKP). Rapa treatment induces dimerization to form an active Cre recombinase (Fig. 1C). Using CRISPR-Cas9, we replaced the endogenous TgBipA gene with the TgBipA coding sequence (CDS) C-terminally tagged with Flag and Ty1 epitopes, and a drug selection cassette flanked by LoxP sites. Accurate integration into the TgBipA locus was confirmed by genomic PCR (Fig. S2A). This line was then modified to express the apicoplast membrane marker APT1 fused to EmeraldFP (EmFP) under the control of the APT1 promoter from the dispensable UPRT locus (referred to as TgBipA-iKO:APT1-EmFP hereafter) (Fig. S2B) (21, 59). To determine the localization of TgBipA, an immunofluorescence assay (IFA) was performed on this line using anti-Ty1 and anti-Cpn60 antibodies that recognize the TgBipA epitope tag and an apicoplast lumen protein, respectively (32). We observed co-localization between TgBipA and Cpn60 signals, surrounded by the APT1-EmFP signal, showing that TgBipA localizes to the apicoplast lumen (Fig. 1D).

### TgBipA is essential to parasite survival

**Fig 2:**
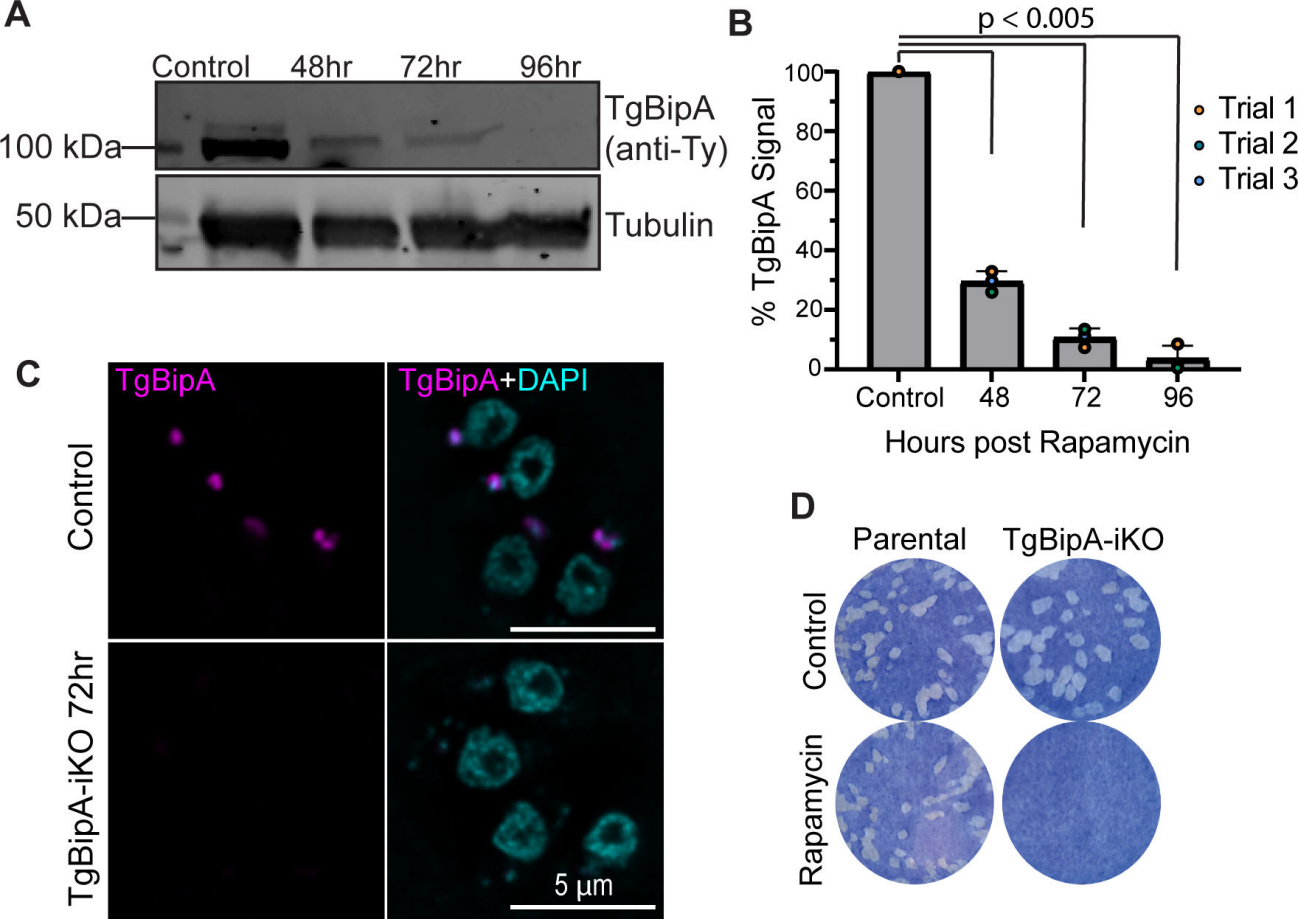
TgBipA is essential for parasite survival. (**A**) Western blot with anti-TY (TgBipA) and anti-tubulin (loading control) antibodies on parasite lysates harvested at 48, 72, and 96 h post-rapamycin treatment, along with control. TgBipA runs at 110 kDa, smaller than the 140 kDa predicted for the full-length protein, suggesting TgBipA is proteolytically processed. (**B**) TgBipA band intensities from (**A**) were quantified relative to the tubulin loading control. Mean and individual values from three biological replicates were plotted. Significant *P* values are indicated (student’s paired t-test). (**C**) IF of TgBipA-iKO parasites treated with DMSO (control) or rapamycin (KO) stained with anti-TY antibodies (TgBipA; magenta) and DAPI (nucleus). TgBipA is undetectable 72 h after rapamycin treatment. Images are maximum intensity Z-projections of deconvolved images. Scale bar = 5 µm. (**D**) Plaque assay of DiCre:T2A:CAT parental line and TgBipA-iKO line treated with DMSO or rapamycin prior to assay initiation. No plaques form upon TgBipA knockout, indicating it is an essential protein.

To determine the efficiency of TgBipA knockout after rapa treatment, we activated the DiCre recombinase by treating parasites with 50 nM rapa for 4 h, and the efficiency of TgBipA excision from the genome was examined by genomic PCR on samples collected 48, 72, and 96 h after rapa treatment. PCR using genomic DNA isolated from TgBipA-iKO shows the integration of the LoxP-flanked TgBipA CDS at the TgBipA genomic locus. PCR on genomic DNA isolated from TgBipA knockouts shows high levels of recombination. No products were produced using primers that bind to the TgBipA CDS (Fig. S3A). An examination of the TgBipA expression levels by western blot shows a predominant band of 100 kDa in control parasites, the predicted size of TgBipA lacking the 400 amino acid N-terminal extension. After 48, 72, and 96 h of rapa treatment, there was a 65%, 92%, and 97% decrease in TgBipA protein levels, respectively, relative to control (Fig. 2A and B; Fig. S3B). In control samples, a fainter band at ~125 kDa is observed, which is smaller than the predicted size of the full-length protein at ~140 kDa. This band could represent pre-mature TgBipA after removal of the SP. Since TgBipA does not contain a predicted SP or apicoplast targeting motif, the sites of TgBipA cleavage are unknown. Consistent with our western blots, TgBipA protein levels were undetectable by immunofluorescence 72 h after rapa treatment (Fig. 2C). Importantly, we do not observe any TgBipA KO parasites with TgBipA levels equivalent to the control, indicating that excision occurs in all TgBipA knock-in parasites (Fig. S3C). To determine whether TgBipA was essential for parasite survival, a plaque assay was performed using the DiCre parental line and TgBipA-iKO parasites treated with DMSO or rapa. TgBipA-iKO parasites treated with rapa failed to form plaques, indicating an essential role of TgBipA in the parasite lytic cycle (Fig. 2D).

### TgBipA is required for apicoplast maintenance

**Fig 3:**
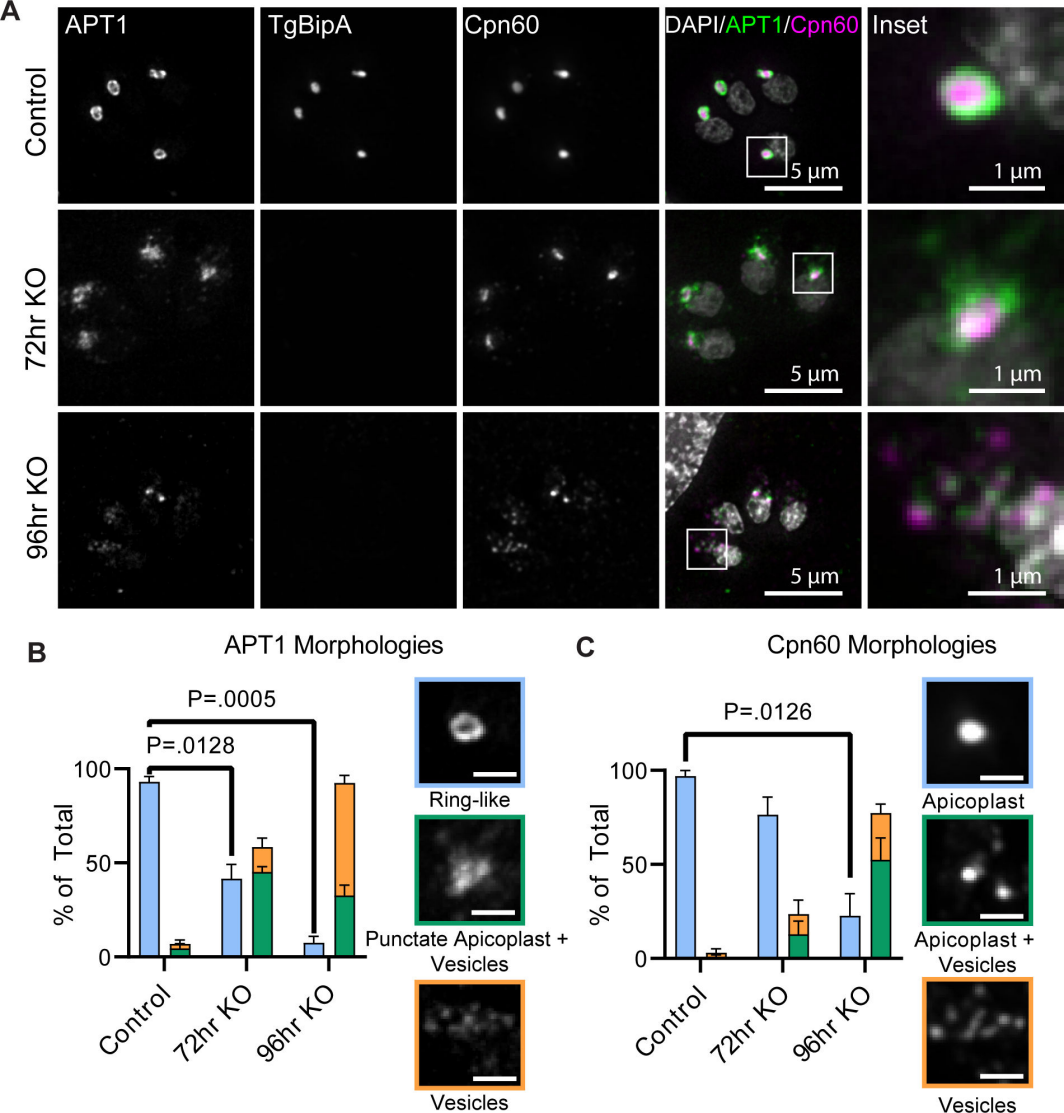
TgBipA KO disrupts apicoplast morphology. (**A**) Immunofluorescence comparing TgBipA-iKO::APT1-GFP and ΔTgBipA:: APT1-GFP parasites 24 h after invasion and 72 h, and 96 h after rapamycin treatment. TgBipA and Cpn60 were visualized using anti-TY and anti-Cpn60 antibodies, respectively. White boxes denote the inset region. In 96-h KO images, the brightness was adjusted to show fainter punctate. Images are maximum intensity Z-projections of deconvolved images. Scale bar = 5 µm, inset = 1 µm. (**B**) Quantification of APT1 and (**C**) Cpn60 morphologies. Data represent three independent experiments with *n* > 100 parasites. Examples of each phenotype are shown in colored boxes, matching the phenotype bar chart color. Significant *P*-values from student’s paired t-test are indicated.

As TgBipA localizes to the apicoplast and is essential, we investigated whether loss of TgBipA impacted apicoplast morphology by examining the localization of APT1 and Cpn60 in interphase parasites. In control parasites (TgBipA-iKO::APT1-EmFP; untreated), a clear ring-like structure of APT1 is observed around the Cpn60 and TgBipA signals (93% ± 2.9% of parasites) (Fig. 3A and B). 72 h after rapa treatment, APT1 appeared ring-like in only 42% ± 7.5% of parasites. In 45% ± 2.8% of parasites, APT1 appears as a puncta with several vesicle-like structures in proximity to the APT1 and Cpn60 puncta. 13% ± 4.8% of parasites contain only vesicle-like structures. At the 96-h time point, the disrupted morphology becomes more predominant, with 60% ± 4% of parasites containing only vesicle-like structures. Additionally, apicoplast DNA could be visualized using DAPI in 85% ± 5.1% of controls compared to just 30% ± 7.7% rapa-treated parasites grown for 96 h after knockout (Fig. S3D). For localization of the lumen marker Cpn60, we see a similar trend to that observed for APT1. 97% ± 2.9% of untreated parasites showed a single bright punctate, consistent with the expected apicoplast lumen localization. This percentage dropped to 76% ± 9.5% 72 h after knockout, while 13% ± 7% contained faint Cpn60 vesicles in addition to the apicoplast. 11% ± 7.3% did not contain a discernible apicoplast, but multiple vesicles were visible. After 96 h of rapa treatment, the Cpn60 localization is further disrupted with 52% ± 11.7% of parasites containing dim Cpn60 vesicles only (Fig. 3A and C). We did not observe any parasites completely lacking APT1 or Cpn60 signals. Notably, the disruption in Cpn60 appears to lag behind that of APT1, indicating temporal differences in how membrane and lumen proteins are disrupted after a TgBipA knockout.

### Loss of TgBipA does not affect apicoplast inheritance

**Fig 4:**
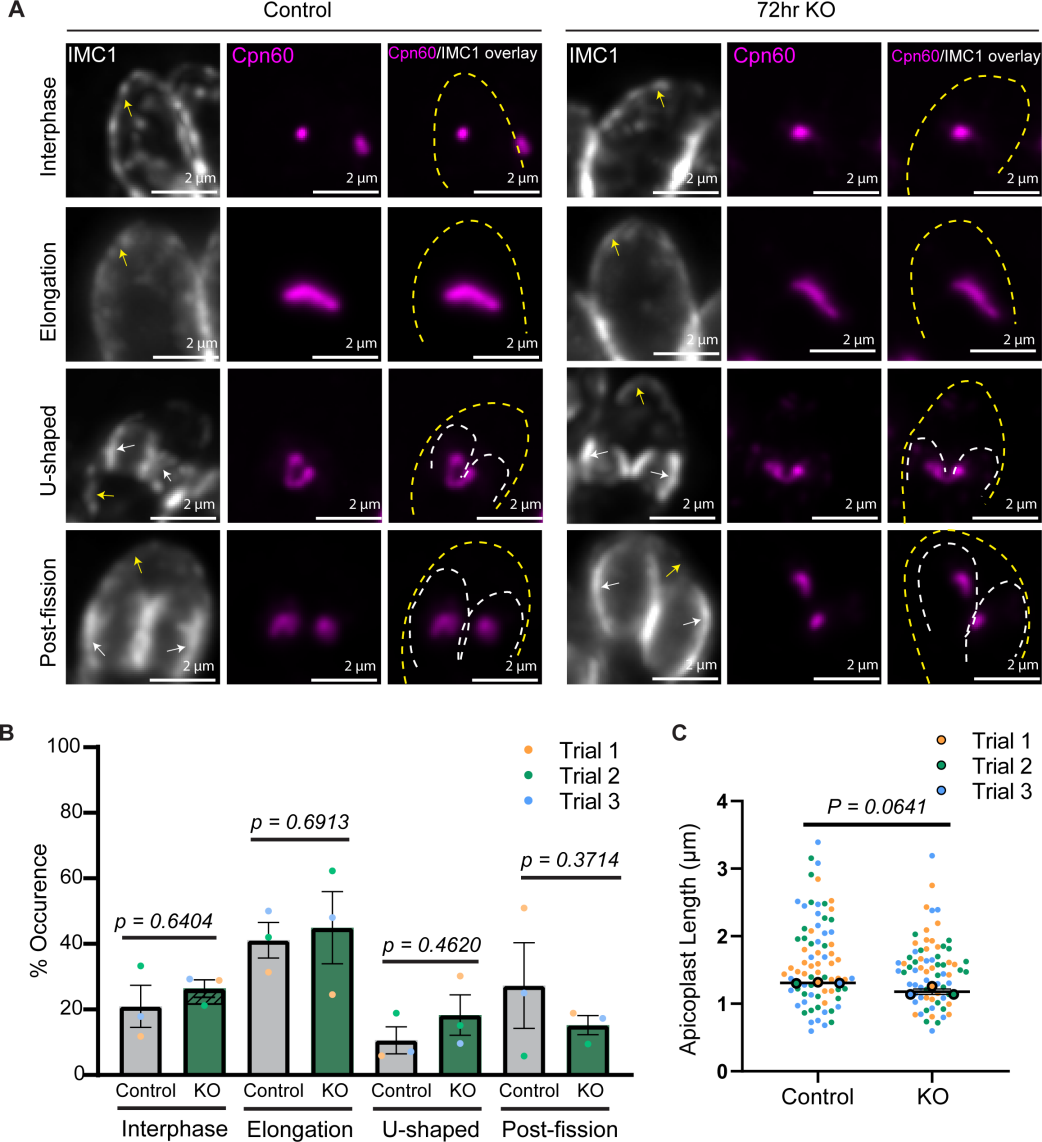
Loss of TgBipA does not disrupt apicoplast inheritance. (**A**) Apicoplast morphology throughout the cell cycle in TgBipA-iKO::APT1-GFP and ΔTgBipA::APT1-GFP (72-h time point) parasites. Apicoplast visualized through anti-Cpn60 antibody (magenta). Periphery of mother (yellow arrows and dashed lines) and daughter (white arrows and dashed lines) parasites visualized using an antibody against inner membrane complex 1 (IMC1) (gray). Images are maximum intensity Z-projections of deconvolved images. Scale bar = 2 µm. (**B**) Percent of parasites in each stage of apicoplast division in control and TgBipA knockout parasites. Data from three independent experiments, *n* > 50 apicoplast. Dashed region of interphase KO denotes parasites with vesicular apicoplast morphology as described in Fig. 3. Error bars represent standard error of means. *P*-values determined by a two-tailed paired student’s t-test. (**C**) Graph showing lengths of apicoplast during the elongation phase of division in TgBipA-iKO::APT1-GFP and ΔTgBipA::APT1-GFP parasites. Data from three independent trials, n of 20. *P-*values from student’s paired t-test.

As we observed a defect in APT1 localization beginning 72 h after TgBipA knockout, we next investigated whether this defect was due to a disruption of apicoplast inheritance. Apicoplast division is a multi-step process: early in cell division, the apicoplast elongates and then associates with the duplicated centrosomes in an ATG8 and actin-dependent manner (31, 60–65). As the daughter cytoskeleton is constructed, the centrosomes and apicoplast ends migrate into the daughter parasites, which results in the apicoplast forming a u-shaped structure (31). DrpA-mediated apicoplast fission results in each daughter inheriting a single apicoplast (66). After 72 h of rapa treatment, there were no differences in the percentage of parasites at each of these stages of division (Fig. 4A and B). In addition, there were no differences in the length of elongated apicoplast in control and TgBipA knockout parasites (1.64 ± 0.01 µm compared with 1.47 ± 0.09 µm) (Fig. 4A and C). Later in the division cycle, the apicoplast was successfully inherited by daughter parasites.

### Loss of TgBipA results in the accumulation of APT1 vesicles

The apicoplast membrane appears less defined upon loss of TgBipA. To better understand this altered membrane morphology, we performed live-cell imaging using the TgBipA-iKO:APT1-emFP line transiently expressing the apicoplast lumen marker FNR fused to RFP. In control interphase parasites, APT1 has a characteristic ring-like structure which surrounds FNR-RFP, with few observable APT1 vesicles (Fig. S4A; Video S1). At 72 h post-rapa treatment, we observed interphase parasites with a disordered APT1 signal surrounding the FNR-RFP signal, with an accumulation of APT1 vesicles surrounding these structures (Fig. S4B; Video S2). Thus, the “punctate” appearance of the apicoplast is due to a combination of a decrease in apicoplast size and accumulation of adjacent vesicles. Consistent with our fixed imaging, we did not observe localization defects in FNR 72 h after knockout. However, beginning at 96 h, we do observe FNR vesicular structures in the apical region of the parasite which do not colocalize with the APT1 vesicles (Fig. S4C; Video S3), consistent with fixed cell data (Fig. 3).

### Accumulated NEAT membrane vesicles have an ER origin

We next wanted to identify the origin of the apicoplast vesicles accumulating in the TgBipA KO. We postulated that these vesicles could be due to either an accumulation of ER-derived vesicles (19, 67, 68), or a destabilization of the apicoplast leading to shedding of APT1-labeled membrane through an unknown pathway. To determine which was occurring, we expressed Halo-tagged APT1 from the UPRT locus in the TgBipA-iKO line (31) (TgBipA-iKO line::APT1-Halo ) (Fig. S5A) and used a pulse-chase method to differentially label existing and newly synthesized APT1 protein with cell-permeable HaloTag fluorescent ligands (31). Differentiating between existing and newly synthesized proteins relies on saturation of existing halo epitopes with halo ligand. To confirm that this was achieved in our experimental parameters, TgBipA-iKO line::APT1-Halo parasites were grown for 24 h and then pulse labeled with TMR ligand for 30 min. Cells were washed and then immediately labeled with JF646 halo dye for 30 min, washed, and immediately imaged (Fig. S5B). In all parasites, TMR strongly labeled the apicoplast, while JF646 was absent from the apicoplast, indicating that the TMR label saturates the Halo protein. Thus, in subsequent experiments, JF646 labeling is indicative of new protein synthesis.

**Fig 5:**
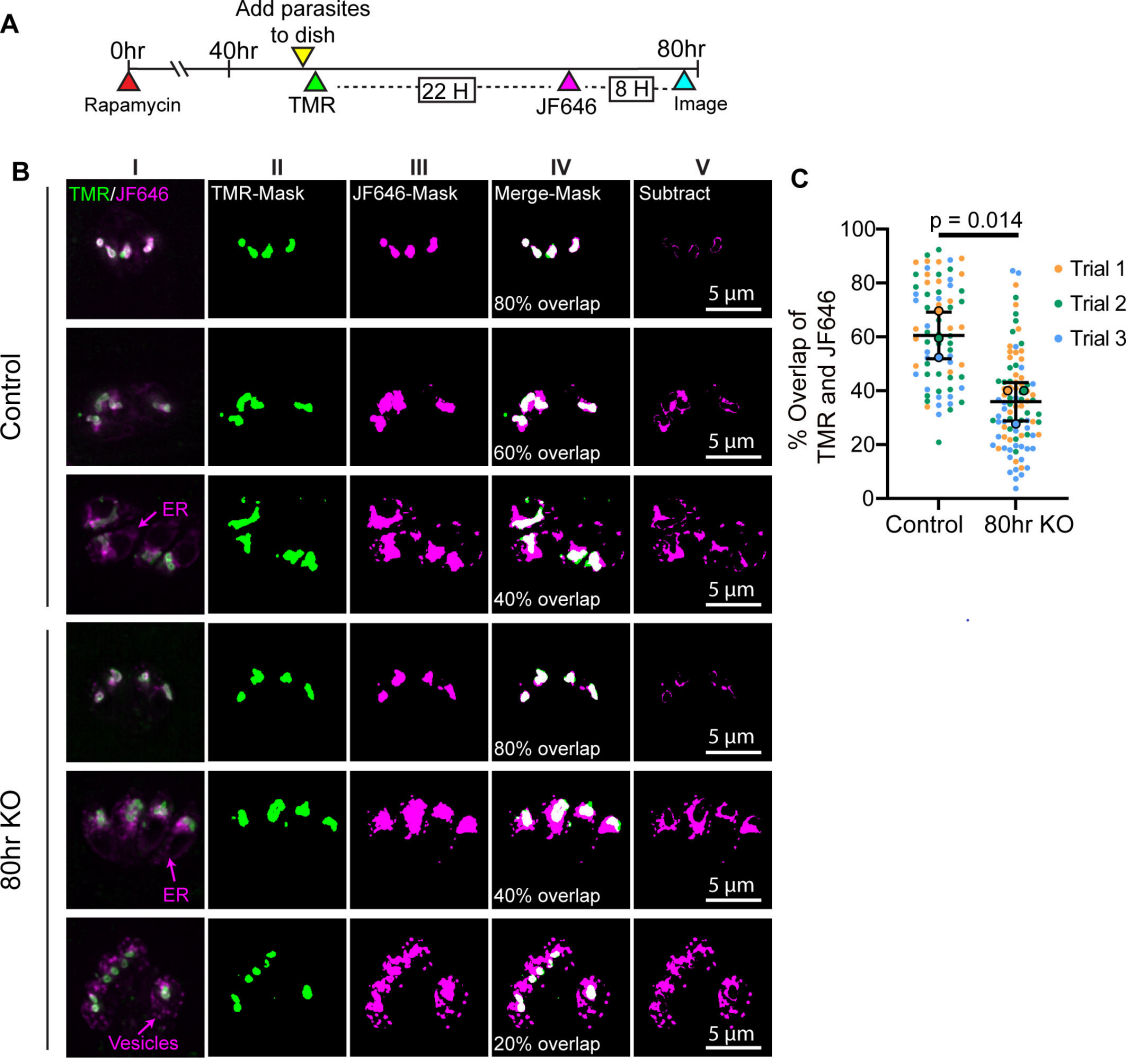
TgBipA KO leads to accumulation of ER-derived vesicles labeled with NEAT membrane proteins. (**A**) Timeline for pulse-chase assay to visualize APT1-Halo trafficking. (**B**) TgBipA-iKO::APT1-Halo and ΔTgBipA::APT1-Halo parasites labeled with TMR and JF646 dyes as outlined in (**A**). In all parasites, Halo-TMR (green; pulse 1) had an apicoplast localization. JF646 (magenta; pulse 2) had a variable distribution and variability in overlap of JF646 and TMR dyes. Column I: maximum intensity projections of deconvolved images. Columns II and III: TMR and JF646 images visualized as binary images. Column IV: Merge of binary TMR and JF646 images. Percent overlap is indicated. Column V: Signal from binary JF646 minus binary TMR signal (Subtract). Scale bar = 5 µm (**C**) Percent of JF646 overlapping with TMR in images from B (see Materials and Methods for details). Mean overlap from each experiment is indicated with the large circles, and individual values are indicated with small circles. Significant *P*-values from Student’s paired t-test. Data from three independent trials, n of 20–30 vacuoles.

With this system, we sought to determine the source of vesicles that accumulate in the cytosol after TgBipA disruption. After 48 h of rapa treatment, parasites were added to Mattek dishes, grown for 2 h and then pulse labeled with TMR-Halo ligand, grown for a further 22 h before pulse labeling with JF646, washed, and imaged after an 8-h outgrowth (Fig. 5A). APT1-TMR was predominantly localized to the apicoplast in both control and knockout conditions, indicating APT1 protein is inherited by daughter apicoplast during division (Fig. 5B; green). There was significant variability in the localization of APT1-JF646. In some parasites, JF646 was almost exclusively localized to the apicoplast, and there was significant overlap between the JF646 and TMR signals (Fig. 5B; top panel shows an 80% overlap). In other parasites, the JF646 signal localized to the ER, as indicated by colocalization with an ER marker (Fig. S5C), or in cytosolic vesicles, indicative of protein that is newly synthesized and trafficking to the apicoplast (Fig. 5B; third panel 40% overlap). The mean overlap between TMR and JF646 in control parasites is 60% ± 8.67% (Fig. 5C). After 80 h of TgBipA knockout, the mean overlap between the two fluorophores was significantly reduced to 36% ± 7.1%, and APT1-JF656 was visible in cytosolic vesicles and in the ER (Fig. 5B and C). The accumulation of APT1-JF646 vesicles in the TgBipA knockout and the absence of APT1-TMR in vesicles indicate that loss of TgBipA disrupts APT1 trafficking and leads to the accumulation of newly synthesized protein in vesicles in the cytosol. To validate this trafficking defect, we investigated whether loss of TgBipA resulted in an accumulation of immature NEAT proteins (34, 36). Western blots were performed on control samples and those grown for 48, 72, and 96 h after rapa treatment using anti-Cpn60, anti-TY (TgBipA), and anti-tubulin as loading controls. In control 48- and 72-h samples, almost all Cpn60 is in the mature cleaved form (mCpn60) with virtually no uncleaved pre-mature Cpn60 (pCpn60) detectable. Accumulation of preCpn60 is observed at the 96-h time point (Fig. S6A).

### Apicoplast translation inhibition and TgBipA knockout results in similar phenotypes

All bacterial trGTPases interact with the ribosome, including bBipA, and thus it is possible that TgBipA might be stably associated with ribosomal components (46, 47, 69). Thus, we performed Flag immunoprecipitation on lysates from TgBipA-iKO parasites and RH parasites as a control to identify TgBipA interacting proteins. While we successfully purified TgBipA from iKO lysates (Fig. S7A), we did not identify any ribosomal components that were significantly enriched in this pulldown compared to controls (Table S4). Besides TgBipA, the only protein significantly enriched in our iKO lysates was a DnaJ domain-containing protein (TgME49_258390). While this protein does have a predicted apicoplast localization (53), it was enriched only 2.2-fold compared to the control. Since this value was only marginally above the twofold enrichment requirement, this protein was not investigated further. In the LC MS-MS analysis of TgBipA, all the identified peptide fragments corresponded to amino acids 413 or greater, providing evidence that the N-terminal region contains the apicoplast targeting motif, which is proteolytically processed during trafficking (Fig. S7B).

**Fig 6:**
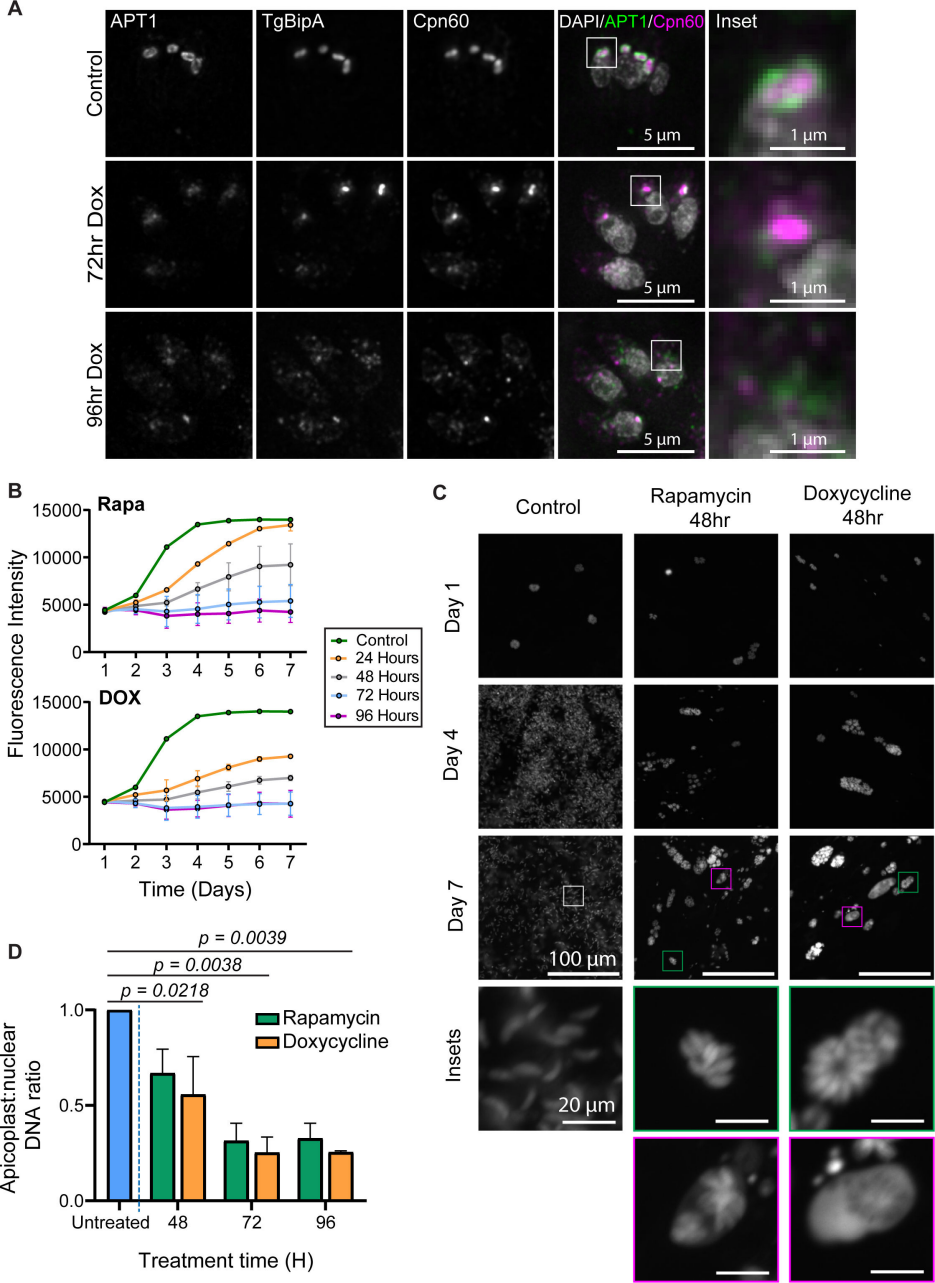
DOX-treated parasites and ΔTgBipA parasites display similar phenotypes. (**A**) Immunofluorescence time course of TgBipA-iKO::APT1-EmFP parasites 24 h post-invasion. Untreated parasites were compared to parasites treated with DOX for 72 and 96 h. TgBipA and Cpn60 were visualized using anti-TY and anti-Cpn60 antibodies, respectively. White boxes denote the inset region. Brightness of 96-h KO images was adjusted to show a fainter punctate signal. Scale bar = 5 µm, inset = 1 µm. (**B**) Spectrophotometer fluorescence intensity readings of TgBipA-iKO::GFP parasites were taken daily for 7 days. The growth rate of control parasites was compared to parasites treated with rapamycin, 24, to 96 h prior to the start of the experiment, or parasites grown continuously in DOX for 24–96 h prior to the start of the experiment. Data from two independent experiments with technical triplicate. (**C**) Images of wells from control and 48-h rapamycin or DOX samples on days 1, 4, and 7. Green box denotes a healthy parasite vacuole with a cytosolic emFP signal, magenta boxes denote a vacuole with an emFP signal in the PV. Scale bar = 100 µm. Inset = 20 µm. (**D**) qPCR to determine relative copy numbers of apicoplast and nuclear genomes at control, 48-, 72-, or 96-h time points. Performed in duplicate with technical triplicate. Error bars represent the standard error of the means. Statistical analysis performed using two-way ANOVA followed by post hoc Bonferroni’s multiple comparisons test

As an alternative approach to address a possible role for TgBipA in apicoplast translation, we compared the TgBipA KO phenotypes to parasites treated with 1 µM DOX. At this dosage, DOX specifically blocks apicoplast translation without inhibiting mitochondrial translation (39, 40). After 72 h of continuous DOX treatment, we observed a disordered APT1 signal, along with an accumulation of APT1 and Cpn60 vesicles (Fig. 6A). At 96 h of continuous treatment, APT1 and Cpn60 were found in punctate structures in the cytosol. Thus, DOX treatment and loss of TgBipA result in similar apicoplast morphology defects (Fig. 3). However, DOX treatment resulted in a greater accumulation of immature NEAT proteins when compared to TgBipA knockout at similar time points (Fig. S6A).

### TgBipA KO and DOX-treated parasites have similar growth rates

Next, we compared the impact of TgBipA knockout and DOX treatment on parasite growth. In the TgBipA-iKO parasite line, an EmeraldFP (EmFP) expression cassette was inserted into the UPRT locus (TgBipA-iKO::EmFP) (Fig. S5A). 24, 48, 72, and 96 h prior to seeding, parasites were pulse treated with rapa or cultured continuously in DOX and then seeded into human foreskin fibroblast (HFF) monolayers in a 96-well plate. GFP fluorescence was then measured with a spectrophotometer daily for 7 days and growth rates compared (Fig. 6B). In addition, the wells were imaged with epifluorescence microscopy to investigate parasite vacuole health (Fig. 6C; Fig. S8). In untreated parasites, we see an increase in fluorescence on days 1–4, followed by a plateau. This coincides with full lysis of the host cell monolayer observed via microscopy (Fig. 6C). For parasites that were drug treated 48 h prior to the start of the growth assay, we see a ~35% and ~50% decrease in growth, for rapa and DOX treatments, respectively, on day 7 of the growth assay (9 days after drug treatment). When these wells were visualized by microscopy, we saw a mixture of healthy-looking vacuoles containing GFP-positive parasites in a rosette pattern (Fig. 6C; green inset box) and unhealthy vacuoles where GFP had leaked into the PV (Fig. 6C; magenta inset box). Almost no growth is observed when the assay was started 72 and 96 h after treatments (Fig. 6B). No treated parasites form plaques in a plaque assay, which indicates that plaque assays are an imprecise method of assessing parasite growth (Fig. 6B; Fig. S6B). Thus, TgBipA KO- and DOX-treated parasites display similar growth kinetics; however, TgBipA knockout growth defects lagged behind DOX treatment, which we hypothesize is due to the lag between the genomic excision of TgBipA and protein turnover.

### Loss of TgBipA results in apicoplast genome replication defects

One of the earliest markers of apicoplast translation inhibition after DOX or clindamycin treatment is a reduction in apicoplast genome copy number due to failure in apicoplast genome replication in the first lytic cycle (45, 48). To determine whether this phenotype was observed in TgBipA knockouts, we used qPCR to determine the relative copy number of the apicoplast localized gene *TufA* to the nuclear gene *Act1* after rapa and DOX treatments (45, 48, 70) (Fig. 6D). For each of the treatment conditions, we saw a ~50% reduction in apicoplast genome copy number at 48 h post-treatment; this was further reduced to ~70% at 72 and 96 h. Thus, loss of genome copy number is the first observable defect after loss of TgBipA or DOX treatment, occurring at the 48-h time point prior to defects in NEAT trafficking. Notably, at this time point, TgBipA has yet to fully deplete from cells (Fig. 2).

### TgBipA exhibits GTPase activity *in vitro*

**Fig 7:**
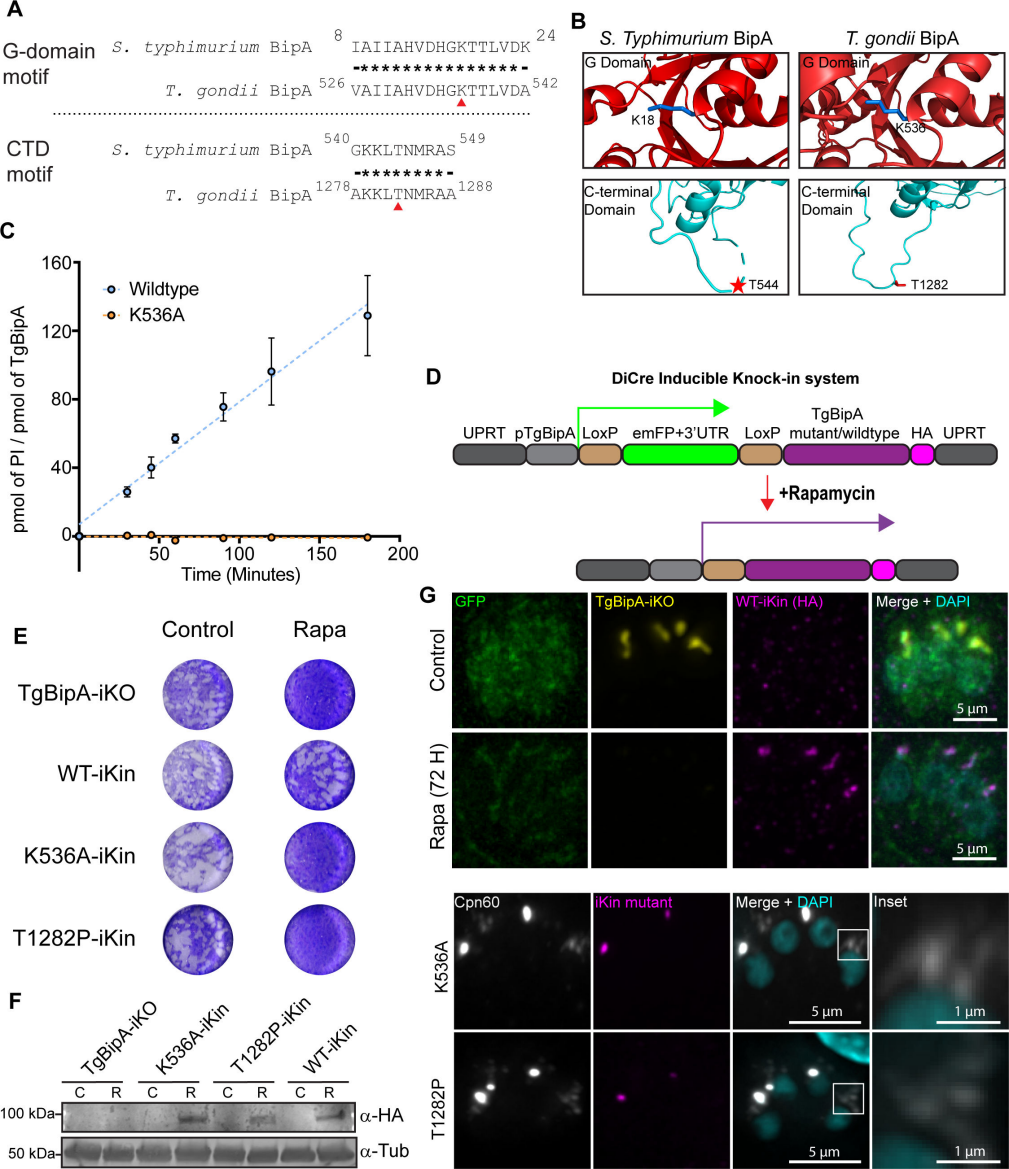
Conserved residues required for GTP hydrolysis and ribosome binding are essential for TgBipA function. (**A**) Amino acid sequence comparison between the G1 motif and a C-terminal loop of bBipA and TgBipA. Conserved residues are denoted by asterisks. Mutated residue denoted by red triangle. (**B**) PDB and AF structures of G and CTD domains of bBipA and TgBipA, respectively, K18/K536 in blue, and T543/T1282 in red. (**C**) Absence of GTPase activity in the TgBipA K536A compared to WT protein was determined by a malachite green end point assay, which detects levels of free phosphate in solution. Error bars represent the standard deviation. (**D**) Schematic of the inducible knock-in (iKI) expression system. The UPRT gene was replaced with emFP, which is expressed using the TgBipA promoter, flanked by LoxP sites. Upon rapamycin treatment, emFP is excised, and TgBipA WT or mutant is expressed. (**E**) Plaque assays performed with TgBipA-iKO (parental), TgBipA WT-iKin, K536A-iKin, and T1282P-iKin parasite lines. (**F**) Anti-HA western blot analysis of TgBipA-HA-iKin^WT/mut^ expression in control (C) parasites and 48 h after rapamycin treatment (R). Tubulin was used as a loading control. (**G**) *Upper:* Immunofluorescence of WT-iKI rescue line in control and rapamycin-treated conditions. TgBipA-iKO visualized by anti-TY antibody (yellow) and WT-iKI visualized by anti-HA antibody (magenta). *Lower:* K536A-iKI and T1282P-iKI lines 96 h after rapamycin treatment. White box denotes the inset region. Scale bar = 5 µm, Inset = 1 µm.

As stated previously, the G-domain of TgBipA is 55% identical to its bacterial counterpart. To determine whether TgBipA has GTPase enzymatic activity, recombinant TgBipA without the N-terminal apicoplast trafficking motif (amino acids 518–1,333) was expressed and purified from *E. coli* (Fig. S9). Utilizing a malachite-green endpoint assay, we confirmed TgBipA’s ability to hydrolyze GTP, suggesting it is a functional GTPase (Fig. 7C). One of the recognizable features of any G-protein is the G1-motif, ^530^AHVDHGKT^537^ in TgBipA, that is identical to the bBipA G-domain (Fig. 7A and B). In Ras, substitution of the lysine in this motif causes severely reduced GTPase activity because the protein cannot properly coordinate the guanine nucleotide or a Mg^2+^ in its active site, generating an inactive GTPase (71). A similar substitution in TgBipA, K536A, resulted in GTP hydrolysis rates that fell below the detection level of the malachite green assay (Fig. 7C).

### Creation of a TgBipA knock-in/knock-out system

To investigate whether GTPase activity was required for TgBipA function *in vivo*, we created a rapa-inducible knock-out/knock-in (iKin) system to conditionally express TgBipA point mutants upon knockout of the WT protein (Fig. 7D; Fig. S10A). The UPRT gene was replaced with a LoxP inducible expression cassette. In untreated parasites, the emFP CDS is transcribed under the TgBipA promoter and blocks the transcription of the downstream TgBipA gene. Upon rapa treatment, emFP is excised via Cre-recombinase and the TgBipA CDS is repositioned downstream of the TgBipA promoter, inducing its expression (72). This coincides with the simultaneous excision of the WT TgBipA gene from the TgBipA genomic locus. This approach allowed us to circumvent any potential dominant negative phenotypes that could arise from constitutive expression of the TgBipA mutant. We first investigated the efficiency of this knock-out/knock-in strategy by placing the HA-tagged WT TgBipA into the knock-in locus (TgBipA^WT^-iKin-HA). In untreated parasites, emFP is present in the cytosol, and TgBipA-iKO (Ty-tagged) is expressed, while the TgBipA^WT^-iKin is not expressed (Fig. 7G). After 72 h of rapa treatment, GFP and TgBipA-iKO-Ty expression are not detectable, and TgBipA^WT^-iKin-HA signal is observed at the apicoplast in 98% of parasites (Fig. 7G; Fig. S10B). Expression of TgBipA^WT^-iKin-HA from the UPRT locus rescued the growth defect observed in the TgBipA knockouts (Fig. 7E).

### TgBipA GTPase activity is required for parasite survival

To determine whether GTP hydrolysis is necessary for TgBipA function, we tested whether the expression of TgBipA^K536A^-iKin could rescue the growth inhibition observed in the TgBipA knockout. We additionally tested a Thr1282Pro (T1282P) mutant, as the equivalent mutation in bBipA (T544P) has been shown to disrupt ribosome binding (Fig. 7A and B) (69). Anti-HA IF confirmed that both mutant proteins were localized to the apicoplast, indicating that these mutations did not disrupt trafficking to the apicoplast (Fig. 7F; Fig. S10A and C). However, unlike complementation with the WT, neither mutation could rescue the TgBipA KO growth defect (Fig. 7E). We next performed an anti-Cpn60 IFA to examine apicoplast morphology (Fig. 7G). In both mutants, we observe parasites lacking a Cpn60 puncta that instead contain vesicular Cpn60 signal (Fig. 7G Inset). Thus, the expression of these mutations cannot overcome the apicoplast morphology defects of the knockout. Taken together, the data indicate that K536 and T1282 are essential residues for TgBipA function within the apicoplast.

## DISCUSSION

The essentiality of the *T. gondii* apicoplast stems from its production of essential metabolites. Since most proteins required for these processes are nuclear encoded and trafficked to the apicoplast, the metabolic pathways are dependent on accurate trafficking of NEAT proteins from the ER, protein translocation across four apicoplast membranes, and chaperones and proteases required for protein maturation in the apicoplast lumen. In addition, two proteins encoded by the apicoplast genome, SufB and ClpC, are thought to contribute to metabolism, although their mechanism of action is unknown (23). Synthesis of these proteins relies on apicoplast genome maintenance, and the transcription and translation machinery involved in gene expression and protein production. Here, we describe an apicoplast-localized GTPase, TgBipA, that is essential for parasite fitness and maintenance of the apicoplast.

### The GTPase activity of TgBipA is essential

TgBipA is a homolog of the bacterial translational GTPase BipA (73). Amino acid sequence comparison as well as an AF model of the protein indicates that the core regions of these proteins are analogous, consisting of a GTPase domain, an OB domain, two alpha-beta domains, and the C-terminal domain, its novel fold characteristic of BipA family members (74). Unique to TgBipA are three very large IDRs, distinctive in that two are serine rich and the third arginine rich. Similar to its bacterial counterpart, TgBipA is an active GTPase. Mutating the conserved lysine in the GTP-binding pocket of TgBipA to alanine (TgBipA K536A) results in a loss of GTP hydrolysis *in vitro*. TgBipA K536A cannot rescue a WT TgBipA knockout, indicating that GTPase activity is critical for the function of the protein. In addition, like its bacterial counterpart, TgBipA has low intrinsic GTPase activity. Whether, like prokaryotic trGTPases, the best known example being EF-G, TgBipA’s hydrolysis rate is significantly enhanced by partner binding remains to be determined (75).

TgBipA has an unusually long N-terminal extension of 400 amino acids. However, no canonical SP or apicoplast targeting motif could be identified in this sequence. While there is no predicted targeting motif, transit peptides in *T. gondii* are typically enriched in serine residues (76), and the TgBipA N-terminal extension is 28% serine, which likely facilitates apicoplast trafficking. When mass spectrometry analysis of affinity-purified TgBipA was performed, peptides corresponding to the first 412 amino acids were consistently missing from our analysis, indicating this sequence corresponds to the apicoplast targeting motif that is removed after import to the apicoplast. When analyzed by Western blot, TgBipA runs at 100 kDa, consistent with the mature protein lacking an approximately 400 amino acid N-terminal extension. DOX treatment results in the accumulation of an additional ~125 kDa band, shorter than the predicted ~140 kDa for the full-length protein. This additional band may represent the pre-protein lacking the ER targeting motif.

### TgBipA is essential for apicoplast maintenance

**Fig 8:**
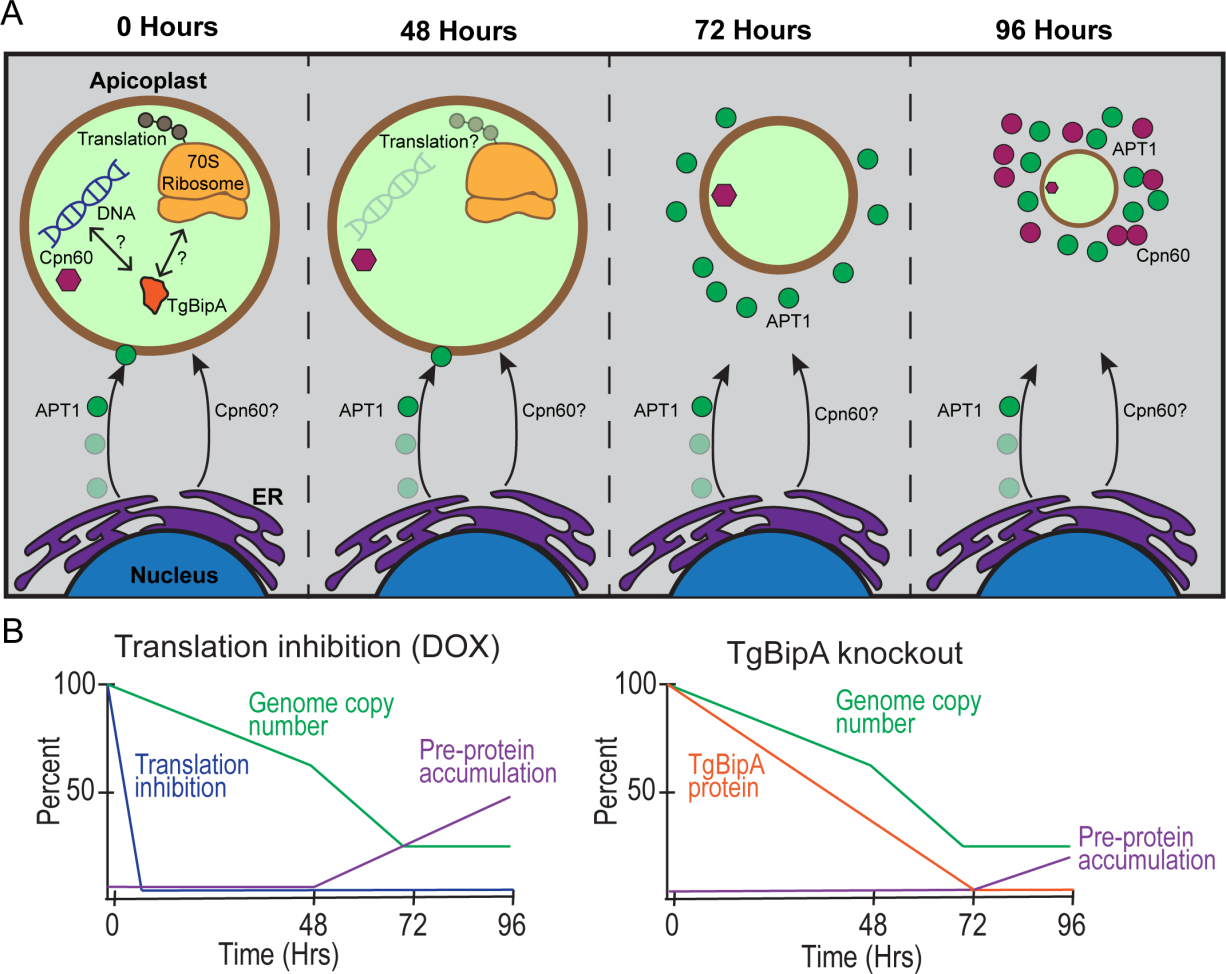
Working model for loss of apicoplast following TgBipA knockout. (**A**) 48 h after TgBipA genomic knockout, there is a reduction in apicoplast genome copy number. 72 h after knockout, there is a loss of APT1 trafficking, and APT1-positive vesicles accumulate in the cytosol. There is a disruption in apicoplast morphology. After 96 h, there is a loss of an observable apicoplast structure. APT1 and Cpn60 vesicles accumulate at the parasite’s apical end. (**B**) Summary of results showing that the observed phenotypes after TgBipA knockout and translation inhibition occur at different time points after treatment.

To gain insight into the mechanism of TgBipA activity, we compared the TgBipA knockout phenotype to parasites treated with translation inhibitors. The overall effects of these treatments were similar, namely, a decrease in genome copy number, accumulation of apicoplast vesicles, defects on NEAT protein proteolytic maturation, and eventually loss of the organelle (Fig. 8A). However, the timing of these events was different in the two conditions, suggesting that TgBipA may not play a direct role in translation (Fig. 8B). First, the decrease in genome copy number occurs 48 h after rapamycin treatment when approximately one-third of TgBipA remains in the cells. A similar defect was observed after apicoplast translation inhibition, consistent with published work. In this case, however, loss of genome copy number is an indirect effect of translation inhibition, which accounts for the 48-h lag time between the start of drug treatment and this phenotypic effect (45, 48). This lag time was not observed in the TgBipA knockout, which may suggest that TgBipA plays a direct role in genome maintenance, rather than translation. Further investigation into, and verification of, the interaction between TgBipA and DnaJ could provide insight into TgBipA’s mechanism of action. Second, both DOX treatment and TgBipA knockout resulted in the accumulation of unprocessed NEAT proteins, indicating a defect in trafficking. Significant accumulation of unprocessed proteins occurs 72 h after DOX treatment, while only a small amount of unprocessed protein has accumulated at the 96-h time point after TgBipA knockout. The defects in apicoplast genome maintenance and trafficking exhibited after translation inhibition highlight the interconnectivity of these cellular processes and the difficulty in assigning mechanistic function to TgBipA based on phenotypic outcomes. The mechanism of TgBipA function needs to be investigated in future studies.

### Vesicle trafficking is disrupted after loss of TgBipA

Following the loss of genome replication, we observed APT1 accumulation in the ER, in newly synthesized, APT1-labeled vesicles adjacent to the apicoplast and a disruption in apicoplast morphology (Fig. 8A, 72 h) as demonstrated through fixed cell (Fig. 3) and live cell (Videos S1 to S3) imaging and pulse-chase assays (Fig. 5). It has previously been proposed that fusion of ER-derived vesicles with the apicoplast would provide the additional lipids necessary for apicoplast expansion prior to division (25, 31). The simultaneous accumulation of vesicles and disruption in apicoplast morphology is consistent with this hypothesis. The pulse-chase assays show that the APT1 protein is inherited by each apicoplast during division, so the disruption in trafficking likely leads to a progressive decrease in protein levels through successive rounds of division. Phenotypic consequences of these trafficking defects would not be observed until membrane protein levels dropped below a certain threshold and could provide an explanation for the delayed growth defects observed in *ΔTgBipA* parasites and after DOX treatment.

24 hours after APT1-labeled vesicles are observed, Cpn60-containing vesicles began to form, and this is accompanied by loss of the apicoplast (Fig. 8A, 96 h). While it has previously been reported that membrane proteins traffic to the apicoplast in ER-derived vesicles, the pathway for NEAT lumen protein trafficking is not clear, as vesicles containing lumen proteins are not observed in healthy, WT parasites (19, 30, 31). Alternative trafficking mechanisms have been proposed, including ribosome docking on the apicoplast outer membrane (23) or rapid transport in vesicles that is difficult to visualize experimentally (30). Regardless of the mechanism in WT parasites, in *ΔTgBipA* parasites, and parasites engineered to survive without the apicoplast (77), membrane and lumen proteins are found in separate vesicle populations that accumulate in the apical region of the parasite. Given that disruption in lumen protein trafficking occurs ~48 h after the reduction in apicoplast genome, this phenotype is likely a downstream, rather than direct, consequence of the TgBipA knockout.

### TgBipA knockout parasites die with variable kinetics

Delayed death of *T. gondii* parasites following perturbation of the apicoplast has been well documented (39, 42, 45, 48, 49). It is typically described as parasite death occurring in the second intracellular growth cycle (~48–96 h), with no growth defects observed in the first intracellular growth cycle (first ~48 h). An extensive time course carried out over the course of 7 days showed that ~48 h after loss of TgBipA and DOX treatment, parasite growth rates were reduced compared to control (Fig. 6). However, a small percentage of parasites formed healthy-looking vacuoles at day 9 after treatment, as these parasites were grown for 2 days prior to the initiation of the 7-day growth assay. It is unclear what accounts for this large variability in the kinetics of parasite death, but this observation demonstrates that death after apicoplast translation inhibition is more delayed than previously appreciated and underscores that translation inhibitors do not demonstrate the optimum killing dynamics for anti-parasitics. While drugs that inhibit apicoplast translation are administered for the treatment of Toxoplasmosis in a clinical setting, these drugs do not rapidly kill the parasite, and prolonged treatment durations are required (78–80). One potential explanation for the continued slow growth of the parasites following apicoplast loss is that the remaining NEAT vesicles may retain low levels of metabolic activity (77, 81).

In conclusion, this study characterizes a new apicoplast-localized GTPase enzyme TgBipA. Loss of this protein results in a decrease in the apicoplast genome, disruption of apicoplast morphology, and trafficking of membrane-associated NEAT proteins, ultimately resulting in parasite death. Future work will focus on identifying TgBipA’s mechanism of action at the apicoplast.

## MATERIALS AND METHODS

### Cell culture

HFFs were grown to confluency in Dulbecco’s modified Eagle’s medium (DMEM) (Fisher Scientific: Catalog#: 11-965-092) supplemented with 1× antibiotic/antimycotic (anti/anti), 1 mM sodium pyruvate, and 10% heat-inactivated fetal bovine serum (FBS) at 37°C with 5% CO_2_. When cells were confluent, media was replaced with DMEM supplemented with 1× anti/anti and 1% heat-inactivated FBS. *T. gondii* parasite lines were continuously passaged in HFFs grown in low serum media.

### Drug treatments

For rapamycin treatment, 10 mM rapamycin (Invitrogen: Catalog#: tlrl-rap-5) in DMSO was diluted 1:100 in molecular biology grade water (Cytiva: Catalog#: SH30538.02) and added to DMEM at 1:2,000 for a final concentration of 50 nM. Parasites were grown in HFFs containing rapamycin media for 4 h before washout. The start time for treatment is considered hour 0 for knockout.

For DOX treatment, 10 mM DOX (Fisher Catalog # BP26531) in DMSO was diluted 1:10 in water and used at a 1:1,000 dilution for a final treatment concentration of 1 µM. Parasites were continuously cultured in DOX-containing media for time points indicated.

For parasite transfections that disrupt the UPRT loci, normal growth media was replaced with media containing 5′-fluo-2′-deoxyuridine (FUDR, Sigma Catalog#: F0503) (10 µM) 24 h after transfection (82).

For parasite transfections which insert the HXGPRT gene, normal growth media was replaced with media containing 25 µg/mL mycophenolic acid (MPA, Sigma Catalog#: M5255-50MG) and 50 µg/mL xanthine (Sigma Catalog#: X0626-5G) 24 h after transfection.

### Parasite transections

All transient and stable parasite transfections were performed using previously described protocols (83–85). Briefly, for transient transfections, 25 µg of plasmid DNA was transfected into 1 × 10^7^ parasites via electroporation. These parasites were grown overnight on MatTek dishes or six-well plates containing confluent HFF monolayers (Mattek Catalog#: P35G-1.5-14-C). To modify genomic loci using CrispR-Cas9, parasites were transfected with 5 µg of plasmid DNA containing a Cas9 expression cassette and guide RNAs and a homologous recombination oligomer (HR oligo) produced by PCR (50 μL). Parasites were grown overnight before switching to drug-containing media. Parasites were subcloned by plating one parasite per well into a 96-well plate (Corning Catalog#: 3585) containing a confluent HFF monolayer and grown undisturbed for 7 days. Positive lines were confirmed via genomic PCR and/or fluorescence microscopy.

### Creation of plasmids and parasite lines

All primers, plasmids, and antibodies in this study are shown in Tables S1 to S3.

For all Gibson assembly reactions, we used a custom master mix using isothermal buffer (100 mM Tris-HCl, pH 7.5, 10 mM MgCl_2_, 0.2 mM dNTPs, 10 mM dithiothreitol [DTT], 5% [wt/vol] PEG 8000, 10 mM β-NAD), 0.0054 U/µL T5 exonuclease (Catalog#: NEB M0663S), 0.034 U/µL Phusion DNA polymerase (Catalog#: NEB M0530S), and 5.34 U/µL Taq DNA ligase (Catalog#: NEB M0208L). 15 µL of custom master mix was used for each reaction with 1–7 µL of DNA fragments incubated at 37°C for 1 h before bacterial transformation. All plasmids were transformed into chemically competent NEB5alpha (New England BioLabs Catalog #C2987I). The sequence of positive clones was verified using whole-plasmid sequencing (Plasmidsaurus). All PCRs were carried out using Q5 high-fidelity DNA polymerase (New England Biolabs Catalog#: M0493L)

#### Creation of TgBipA-iKO parasite line

*Insertion of BipA gRNA into pU6 universal plasmid*. Complementary oligos containing 5′ and 3′ guide RNA sequences (Table S1) were reconstituted to a concentration of 200 µM in molecular biology grade water, oligos were phosphorylated with PNK and then annealed by heating to 95°C and then cooling at a rate of 0.1°C per second. Duplexed oligos were ligated to pU6 universal plasmid (pU6-Universal, AddGene plasmid # 52694), digested with BsaI using T4 DNA ligase (New England Biolabs) as per the manufacturer’s instructions. To create a plasmid containing both 5′ and 3′ BipA guide RNAs, the pU6-5′ BipA plasmid was linearized with KpnI. The 3′gRNA, scaffold, and promoter were amplified by PCR using KpnI-pU6promF and ptub-KpnI-R and then digested with KpnI enzyme. The linearized plasmid and digested PCR product were ligated using T4 DNA ligase (NEB) as per the manufacturer’s instructions.

To create the template for the HR oligo that contains the TgBipA CDS containing Ty1 and Flag epitopes, we first cloned the TgBipA CDS into the pFastBac plasmid (unpublished plasmid). Due to the size of the gene, the CDS was amplified with a series of nested primers (cBipA primers; Table S1). Fragments were then annealed to the linearized pFastBac plasmid containing a Flag epitope using Gibson assembly. The BipA-Flag was then subcloned into a plasmid containing a LoxP site downstream of the HXGPRT selection cassette (86), as illustrated in Fig. 1C. This plasmid (pTKOII-MyoF-LoxP) was digested with AvrII and AflII and plasmid backbone gel purified. TgBipA-Flag was amplified using primers BipA_CreLoxP and Flag-3xTy-R. Note the forward primer contains a LoxP site upstream of the BipA start ATG. 3xTy1 was amplified using primers 3xTy1-F and 3xTy1-R. Plasmid backbone and both PCRs were annealed using Gibson assembly as described above.

#### Creation of pTub-emFP-UPRT parasite line

In the TgBipA-iKO:pTub-emFP-UPRT line, emFP is expressed from the UPRT loci under the control of the tubulin promoter. To create this line, the 5′UPRT-pAPT1-APT1-emGFP-3′UPRT plasmid (Table S2) (31) was digested with AvrII and AflII to remove pAPT1-APT1-emGFP, and the linearized backbone was gel-purified following the manufacturer’s instructions (Qiagen Catalog#: 28706). pTub-emFP-3′DHFR was amplified via PCR (unpublished plasmid, Table S3) with AvrII and AflII overhangs and gel purified and put into the UPRT backbone through Gibson assembly following manufacturer’s instructions (New England Biolabs Catalog#: M5520AA2).

#### Transfection and integration of constructs into the UPRT genomic loci

To create all HR oligos for transfection into the UPRT loci, a 50 µL PCR reaction was performed using UPRT F and UPRT R primers and plasmid DNA template (Table S2). This PCR product was transfected into 1 × 10^7^ TgBipA-iKO parasites along with 5 µg of pSag1::Cas9::U6::sgUPRT plasmid (AddGene plasmid # 54467). FUDR drug selection was carried out as described above. Positive clones were selected and confirmed for successful integration via genomic PCR and fluorescence microscopy (Fig. S2B; Tables S1 and S2).

#### Creation of TgBipA:APT1-emFP-UPRT and TgBipA:APT1-Halo-UPRT parasite lines

The plasmids used to make these lines were previously described (31). Transfection into the UPRT loci was performed as described above.

#### Creation of TgBipA iKI WT parasite line

For the TgBipA iKI lines, mutant or WT TgBipA was expressed from the UPRT loci using the pTgBipA promoter and 3′UPRT UTR. To create this line, TgBipA CDS was PCR amplified from the pTKOII_TgBipA_CDSFlag_LoxP plasmid with overhangs to an HA gblock containing an NheI cut site and overhangs into the pTgBipA promoter with an NheI cut site. pTgBipA promoter was PCR amplified from RH gDNA with TgBipA CDS and 5′UPRT overhangs containing NheI and NdeI cut sites. 3xHA-stop gblock with TgBipA CDS and 3′UPRT overhangs was ordered from IDT as forward and reverse ultramers and annealed as described above. All products were gel-purified following the manufacturer’s instructions. 5′UPRT-pAPT1-APT1-emGFP-3′UTR was digested with AvrII and AflII to remove pAPT1-APT1-emGFP and the linearized backbone was gel-purified following the manufacturer’s instructions. Ligation of PCR products was performed via Gibson assembly to create 5′UPRT_pTgBipA_TgBipA CDS-3xHA_3′UPRT. emFP-3′DHFR was amplified from pTub-emFP-3′DHFR plasmid via PCR using ultramers with LoxP overhangs and homology to pTgBipA promoter and TgBipA CDS, and gel-purified. 5′UPRT_pTgBipA_TgBipA CDS-3xHA_3′UPRT was digested with NdeI and gel purified. LoxP-emFP-LoxP was ligated into 5′UPRT_pTgBipA_TgBipA CDS-3xHA_3′UPRT backbone using Gibson assembly to create 5′UPRT_pTgBipA_Loxp-emFP-3′DHFR-Loxp-TgBipA CDS-3xHA_3′UPRT. This plasmid was transformed into NEBDH5α cells, and positive clones were verified through whole-plasmid sequencing.

#### Creation of TgBipA^K536A^-iKin and TgBipA^T1282P^-iKin parasite lines

For creating iKI rescue lines TgBipA^K536A^-iKin and TgBipA^T1282P^-iKin, 3′-DHFR-Loxp-TgBipA CDS with emFP and 3′UPRT overhangs was amplified from 5′UPRT_pTgBipA_Loxp-emFP-3′DHFR-Loxp-TgBipA CDS-3xHA_3′UPRT in two pieces, with overlap at the mutation site using TgBipA K536A mutant Fwrd/Rev primers to create a base mutation resulting in a K/A amino acid change, and TgBipA T1282P mutant Fwrd/Rev Primers to make the T/P amino acid mutation, and PCR products were gel purified. 5′UPRT_pTgBipA_Loxp-emFP-3′DHFR-Loxp-TgBipA CDS-3xHA_3′UPRT was digested with AflII and HindIII and gel purified to remove 3′DHFR-Loxp-TgBipA CDS-3xHA. PCR products were inserted into the backbone using Gibson assembly.

### qPCR

Parasite genomic DNA was extracted using Qiagen DNeasy Tissue kit (Catalog#: 69504) according to the manufacturer’s instructions. qPCR was performed using iTaq universal SYBR green supermix (Biorad Catalog#: 1725121) on a Biorad CFX96 Real-Time PCR machine using primers in Table S1. Relative DNA quantity was determined using the Pfaffl method (87).

qPCR run information: Following an initial incubation at 95°C for 3 min, 40 cycles of 95°C for 10 s, 55°C for 20 s, 72°C for 20 s were performed.

### Fluorescence growth assay

In total, 6,000 control, DOX- or rapamycin-treated parasites were added to each well of a 96-well plate (Greiner One Catalog#: 655097) in Gibco fluorobrite DMEM (Catalog#: A1896701) supplemented with 1× anti/anti, 1% heat-inactivated FBS, and 4 mM L-Glutamine and containing confluent HFF monolayers. For DOX-treated samples, the assay was performed with media containing 1 μM DOX. GFP fluorescence was measured daily using a Molecular Devices SpectraMax i3x. Each reported reading was an average of 21 measurements taken per well.

### Western blot

Parasites were syringe released, counted, and 6 × 10^7^ parasites were centrifuged at 1,260 × *g* for 4 min. Cells were resuspended in 1× PBS. 10× sample buffer was added to a final concentration of 2× (10× sample buffer: 0.6M Tris-HCl pH 6.8, 700 mM sodium dodecyl sulfate (SDS), 20% glycerol, 0.75 mM bromophenol blue, 2.5 mM 2-mercaptoethanol, and 25 mM dithiothreitol). Samples were boiled for 10 min at 95°C. Samples equivalent to 2 × 10^7^ parasites were run on Bio-Rad 4%–20% gradient gels (Catalog#: 4561094) and transferred to a nitrocellulose membrane. Membranes were blocked in blocking buffer (2% non-fat milk in 1× TBST [0.1M Tris-base pH 7.4, 10% Tween-20, 0.15M NaCl, in water]) for 2 h and incubated with primary antibodies diluted in blocking buffer overnight (Table S3). The membrane was washed three times for 10 min each in 1× TBST and incubated with secondary antibodies according to Table S3 for 60 min. Blot was washed three times and imaged on a LiCor Odyssey Fc with Pierce ECL western blotting substrate (ThermoFisher Cat #: 32209), or on an Odyssey CLx.

### Plaque assays

To determine whether TgBipA is essential for parasite viability, TgBipA-iKO or RH:Dicre:T2A:CAT parasites were treated with rapamycin or an equivalent volume of DMSO, grown for 72 h, and then 600 parasites were plated onto a six-well plate containing a confluent HFF monolayer and grown undisturbed for 7 days. Wells were fixed with methanol cooled to −20°C for 15 min and then stained with Coomassie (53% H_2_O, 40% methanol, 7% acetic acid, 300 µM Brilliant blue R [Sigma-Aldrich B7920]) for 2 h and then destained (53% H_2_O, 40% methanol, 7% acetic acid) for 2 h. TgBipA mutant rescue plaque assays and DOX time course plaque assays were performed as above in a 12-well plate.

### Pulse-chase assay

For Halo-ligand labeling of APT1-Halo, parasites in a monolayer of HFFs in a MatTek dish were incubated with Halo dyes according to the timeline illustrated in Fig. 5. TMR-(Promega G8252) and Janelia fluor 646 Halo dyes (Jf646) (Promega GA1120) were used at a final concentration of 1 µM in DMEM. Janelia fluor 554 (Jf554) (Promega HT1030) was used at a final concentration of 200 nM in DMEM. Incubations were carried out for 30 min. After staining, cells were washed 3× times in DMEM and imaged in fluorobrite DMEM.

### Fluorescence microscopy

Imaging was performed on two microscopes: a DeltaVision Elite microscope system built on an Olympus base with a 100 × 1.39 NA and 60 × 1.42 NA objective in an environment chamber heated to 37°C. This system uses a scientific CMOS camera and DV Insight solid-state illumination module with the following excitation wavelengths: DAPI = 390/18 nm, FITC = 475/28 nm, TRITC = 542/27 nm, and Alexa 647 = 632/22 nm. Single band pass emission filters had the following wavelengths: DAPI 435/48 nm, FITC = 525/48 nm, TRITC = 597/45 nm, and Alexa 647 = 679/34 nm. A Nikon TI-2 microscope system with 100 × 1.45 NA, 60 × 1.42 NA, and 40 × 0.60 NA objectives in an environment chamber heated to 37°C. This system uses an ORCA-Fusion C14440 digital CMOS camera and Lumencor Spectra light engine with the following excitation wavelengths: DAPI = 390/22 nm, FITC = 475/28 nm, Cy3 = 555/28 nm, and Cy5 = 637/12 nm. Single band emission filters had the following wavelengths: DAPI = 432/36 nm, FITC = 515/30 nm, Cy3 = 595/31 nm, Cy5 = 680/24 nm. Image acquisition speeds for the live cell imaging were determined on a case-by-case basis and are indicated in the figure legends. For multiple focal planes, a Z-step of 0.2 μm (fixed and pulse chase) or single Z-slices (live videos) was used. Images in figures represent a maximum intensity Z-slice projection unless stated otherwise. Brightness and contrast are normalized unless noted in the figure legend.

### Immunofluorescence assays

Coverslips were fixed in 4% paraformaldehyde (Electron Microscopy Sciences, Catalog#: 15714) in 1× PBS for 15 min at room temperature. Coverslips were washed 3× times in PBS before permeabilization with 0.25% Triton-X 100 (ThermoFisher Catalog#: 28314) for 15 min. Coverslips were washed 3× times in PBS and blocked in 2% BSA (Fisher Catalog# BP9703) in PBS for 20 min. Antibodies were diluted in PBS according to Table S3, and incubations were carried out for 30 min. Cells were washed three times in 1× PBS washes between primary and secondary antibody incubations. 10 µM DAPI (ThermoFisher Catalog#: D1306) diluted in PBS was added to samples for 10 min, followed by 3× PBS washes. Coverslips were mounted onto slides with Prolong Diamond (ThermoFisher Catalog#: P36970) or Vectashield Plus (Fisher Catalog#: H19002) and imaged after drying overnight (Prolong) or immediately (Vectashield Plus).

#### Live imaging of TgBipA-iKO::APT1_emFP parasites transfected with FNR-RFP plasmid

Control or rapamycin-treated parasites were transfected with an FNR-RFP expression plasmid (Table S2), as described above, and grown for 24 h in MatTek dishes containing confluent HFF monolayers. Prior to imaging, dishes were washed three times in 1× PBS and then imaged in fluorobrite DMEM. Imaged at 3.3 frames per second on a single Z-slice using 25 ms and 5 ms exposure times for APT1 and FNR-RFP, respectively.

### TgBipA co-immunoprecipitation pulldown

Approximately 1 × 10^9^ parasites per condition were pelleted at 1,260 × *g* for 4 min and resuspended in 10 mL of 1× PBS. Cells were pelleted again and resuspended in 5 mL of 1× Lysis buffer (10 mM imidazole, 300 mM KCl, 1 mM EGTA, 5 mM MgCl_2_, 125 µM GTP [Millipore Catalog#: GE27207601], 2 mM DTT, 1% TX-100, 0.5 mM PMSF, 10 µg/mL leupeptin, 10 µg/mL pepstatin, 10 µg/mL aprotinin, 10 µg/mL calpeptin, 1.6 mg/mL benzamidine, 1:200 dilution of protease inhibitor cocktail [Sigma Catalog#: P8340]) and incubated on ice for 10 min. Samples were centrifuged at 17,000 × *g* for 30 min at 4°C. Supernatant was incubated with 25 µL of Anti-FLAG magnetic beads (Fisher Scientific Catalog#: PIA36797) for 1 h on a shaker at 4°C. Beads were washed 10 times with 1 mL of wash buffer (10 mM imidazole, 300 mM KCl, 1 mM EGTA, 5 mM MgCl_2_, 0.100 mM GTP, 2 mM DTT) followed by 2 washes in 1× PBS and stored at 4°C in 1× PBS until analysis by mass spectrometry.

### Image processing, analysis, and statistics

Graphs made in GraphPad Prism as bar charts or superplots (88)

#### Quantification of apicoplast morphologies in interphase parasites

To quantify apicoplast morphology upon BipA knockdown, fields of view containing at least one parasite vacuole with at least two parasites in the vacuole were imaged, scored, and the APT1 signal was categorized based on a ring-like, puncta, or vesicular. Cpn60 was categorized based on a puncta: apicoplast puncta with vesicles or vesicles only. *N* = >100 parasites in each of three independent experiments. Error bars represent the standard error of the mean. Statistical significance was determined using the means of each experiment with a two-tailed Student’s paired t-test.

#### Apicoplast morphology in the cell cycle

To quantify the cell cycle stage of the apicoplast upon BipA knockdown, fields of view containing at least one parasite vacuole with daughter IMC1 signal within a mother IMC1 were imaged. Apicoplast stage was scored using the Cpn60 signal and categorized into interphase, elongated, u-shaped, or post-fission morphologies. *N* = >50 apicoplasts in each of three independent experiments. Error bars represent the standard error of the mean. Statistical significance was determined using mean values from the three independent experiments through a two-tailed Student’s paired t-test.

#### Apicoplast length measurements

To determine whether there was a change in apicoplast length during division, apicoplast length was measured on max intensity Z-projections of images with parasites containing an elongated or u-shaped apicoplast using the Cpn60 signal and the segmented line tool in Fiji. *N* = 25 parasites from three independent experiments. Error bars represent the standard error of the mean. Statistical significance was determined using the means of each experiment with a two-tailed Student’s Paired t-test.

#### Quantification of pulse-chase assay

To quantify the percent of the Jf646 signal which overlaps with the TMR max intensity Z-projections were created. Threshold images were generated using the default thresholding setting in Fiji, then converted to a binary mask, and the mask area was calculated using Analyze Particles tool. The percent overlap of the Jf646 signal with the TMR signal was calculated using the formula:


$$\%\;Overlap=\left(1-\frac{Jf646\;area-TMR\;area}{Jf646\;area}\right)*100$$

*N* = 20–30 vacuoles from three independent experiments. Error bars represent the standard error of the mean. Statistical significance was determined using a two-tailed Student’s paired t-test.

#### qPCR

Statistical significance was determined by using a two-way ANOVA followed by post hoc Bonferroni’s multiple comparisons test.

#### Bleach correction

Time-lapse images and movies were bleached and corrected using the histogram matching method in FIJI.

#### Deconvolution

Deconvolution was performed using Softworx v7.2.2 (DeltaVision Elite) and the Richardson-Lucy method (Nikon Ti-2). All images in figures are deconvolved unless stated otherwise.

#### Expression and purification of TgBipA from bacteria

An N-terminal his-tagged truncated TgBipA construct was ordered from Twist Biosciences by inserting *E. coli* optimized cDNA encoding residues 518–1,333 of TgBipA into a pET28a vector. The Lys536Ala (K536A) variant was constructed using a QuikChange site-directed mutagenesis protocol using primers listed in Table S1. Plasmids were confirmed with Sanger sequencing. WT and K536A mutant recombinant proteins were expressed in *E. coli* BL21(DE3) cells. Cells were grown at 37°C in Luria-Bertani medium supplemented with 30 µg/mL kanamycin to the mid-log phase and induced with 0.5 mM isopropyl β-D-thiogalactopyranoside. The cells were grown an additional 12–15 h at 16°C and harvested by centrifugation (5,000 × *g* for 30 min at 4°C). Cells were washed in 200 mM NaCl, 20 mM Tris-HCl (pH 7.5), pelleted by centrifugation, and stored at −20°C.

Protein purification was done at 4°C. Cell pellets were resuspended in 20 mM HEPES, 20 mM imidazole, 400 mM KCl, 1 mM DTT, 1% glycerol, and lysed by sonication, and the lysate clarified by centrifugation (20,000 × *g* for 30 min at 4°C). The supernatant was then loaded onto a HisTrap FF crude column (Cytiva LifeSciences). TgBipA was eluted with an imidazole gradient from 0.02 to 0.4 M in the same buffer. His-tagged protein was concentrated to <2 mL using an Amicon Centrifugal Filter Unit (Millipore) and applied to a Superdex 200 16/60 prep grade column (Cytiva LifeSciences) equilibrated in 20 mM Hepes, 250 mM KCl, 1 mM EDTA, 1 mM DTT, and 1% glycerol. Fractions were analyzed by SDS-polyacrylamide gel electrophoresis, and those containing protein that was >95% pure were pooled. Protein quantification was done with the Pierce BCA Protein Assay (ThermoFisher; Catalog #23225).

#### GTP hydrolysis endpoint assays

The GTPase activity of TgBipA was measured using a malachite green endpoint colorimetric assay (89). TgBipA (1 µM) was incubated with 3 mM GTP at 37°C for 3 h. To stop the reaction, 30 µL of the reaction mixture was added to 0.8 µL of acidic malachite green solution. After incubation for 30 min at room temperature, color formation was assessed at 660 nm using a ThermoFisher Scientific Genesys 150 UV-visible Light Spectrophotometer. Background GTP hydrolysis rates were determined for 3 mM GTP. Reactions were performed at least two times. Experiments were performed in 20 mM HEPES, 150 mM KCl, 20 mM MgCl, and 1 mM DTT.

## AFTER EPUB

*[This article was published on 9 December 2025 with an error in Victoria L. Robinson's surname. The name was corrected in the current version, posted on 10 December 2025.]*

## SUPPLEMENTAL MATERIAL

Supplemental Figures msphere.00713-25-s0001.docx Figures S1 to S10.

Table S1 msphere.00713-25-s0002.xlsx List of primers used in this study.

Table S2 msphere.00713-25-s0003.xlsx List of plasmids used in this study.

Table S3 msphere.00713-25-s0004.xlsx List of antibodies used in this study.

Table S4 msphere.00713-25-s0005.xls TgBipA mass spectrometry data.

Video S1 msphere.00713-25-s0006.avi Control TgBipA-iKO parasites expressing APT1-emFP and FNR-RFP imaged live for 30 s.

Video S2 msphere.00713-25-s0007.avi ΔTgBipA (72 h after rapa treatment) parasites expressing APT1-emFP and FNR-RFP imaged live for 30 s.

Video S3 msphere.00713-25-s0008.avi ΔTgBipA (96 h after rapa treatment) parasites expressing APT1-emFP and FNR-RFP imaged live for 30 s.

ASM does not own the copyrights to Supplemental Material that may be linked to, or accessed through, an article. The authors have granted ASM a non-exclusive, world-wide license to publish the Supplemental Material files. Please contact the corresponding author directly for reuse.
