# Supplementary material for: An apicoplast-localized GTPase is essential for Toxoplasma gondii survival
Source: mSphere. 2025 Dec 9;11(1):e00713-25. doi: 10.1128/msphere.00713-25 (PMC12838348; doi:10.1128/msphere.00713-25)
Supplement: Supplemental Figures — Figures S1 to S10. [file msphere.00713-25-s0001.docx]

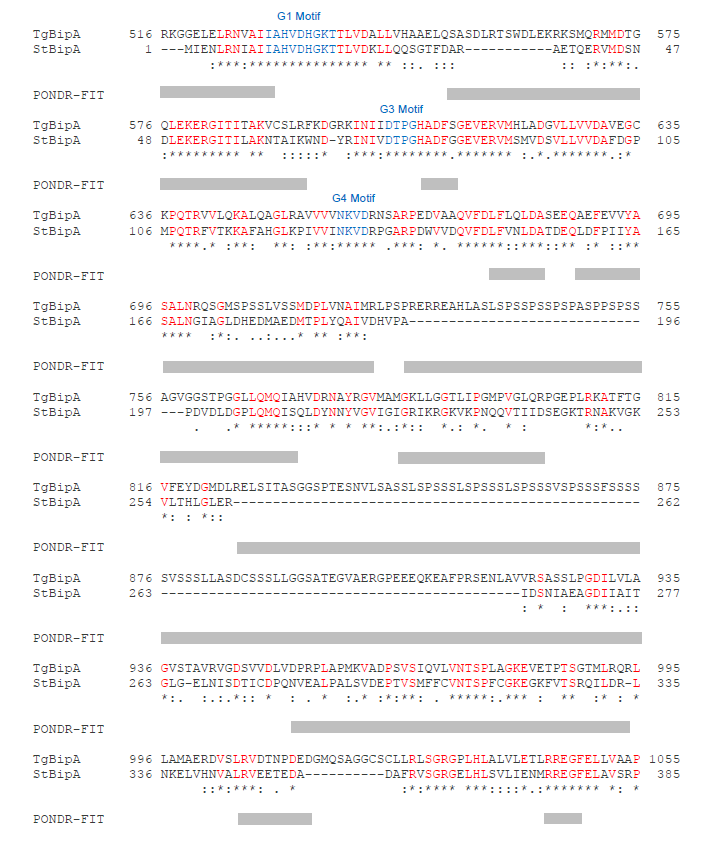


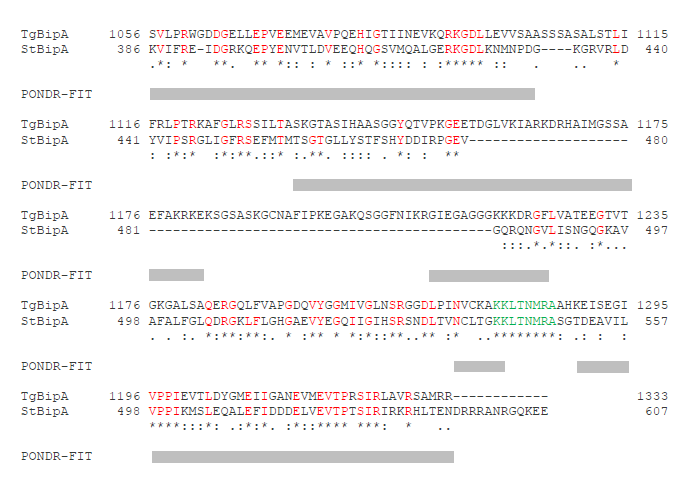


**Figure S1**. Amino acid sequence comparison between TgBipA (TgME49_301380) and *S. enterica* BipA done with the Clustal Omega multiple sequence alignment algorithm^52^. The two proteins are 41% identical with those residues colored red. Residues that are strictly conserved are designated with a star, similar residues with a vertical double dot. The G motifs, defining the G-domain of the protein are highlighted in blue and the conserved loop in the C-terminal domain of the protein, colored green. In addition, the intrinsically disordered residues in TgBipA, predicted using PONDR-FIT^55^, are designated by a grey horizontal line below the amino acid sequences.


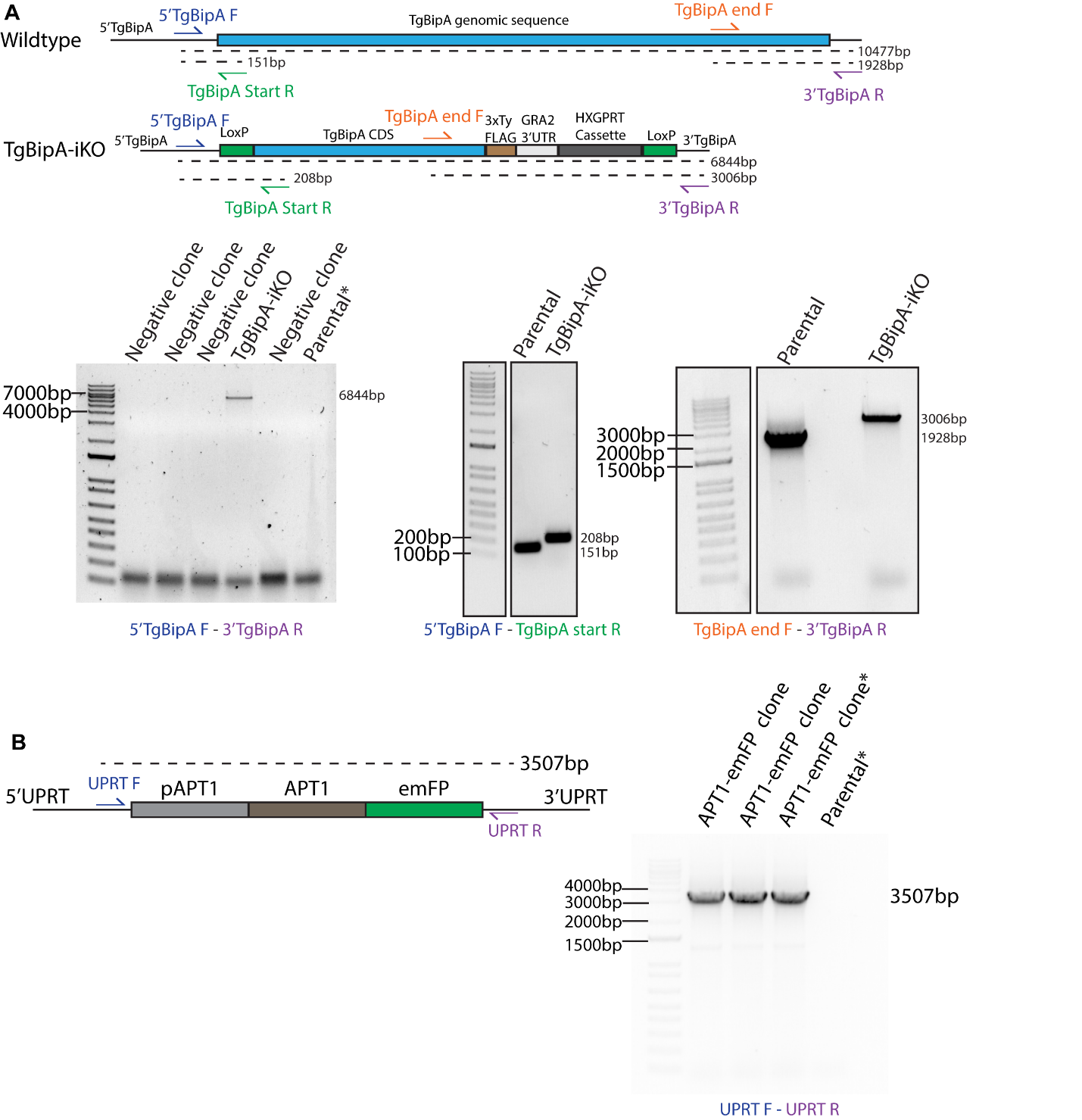


**Figure S2.**

A) *Upper:* Graphic representation of the TgBipA genomic loci in parental and iKO parasite lines respectively. Primer binding sites are indicated. *Lower:* Genomic PCRs for TgBipA-iKO and parental parasite lines. For 5’TgBipA – 3’TgBipA PCR, predicted size of parental loci is 10500bp and yielded no product (Parental* lane). Middle and right gel images are cropped to remove unneeded lanes, cropping denoted by black border. B) *Left:* Schematic of the modified UPRT locus expressing APT1-emFP. *Right:* Genomic PCR confirmation for insertion of pAPT1-emFP into the UPRT locus of TgBipA-iKO parasite line clones. Positive clone used in this study denoted by asterisk. Predicted size for wildtype UPRT loci is 5000 base pairs and yielded no product (Parental* lane).


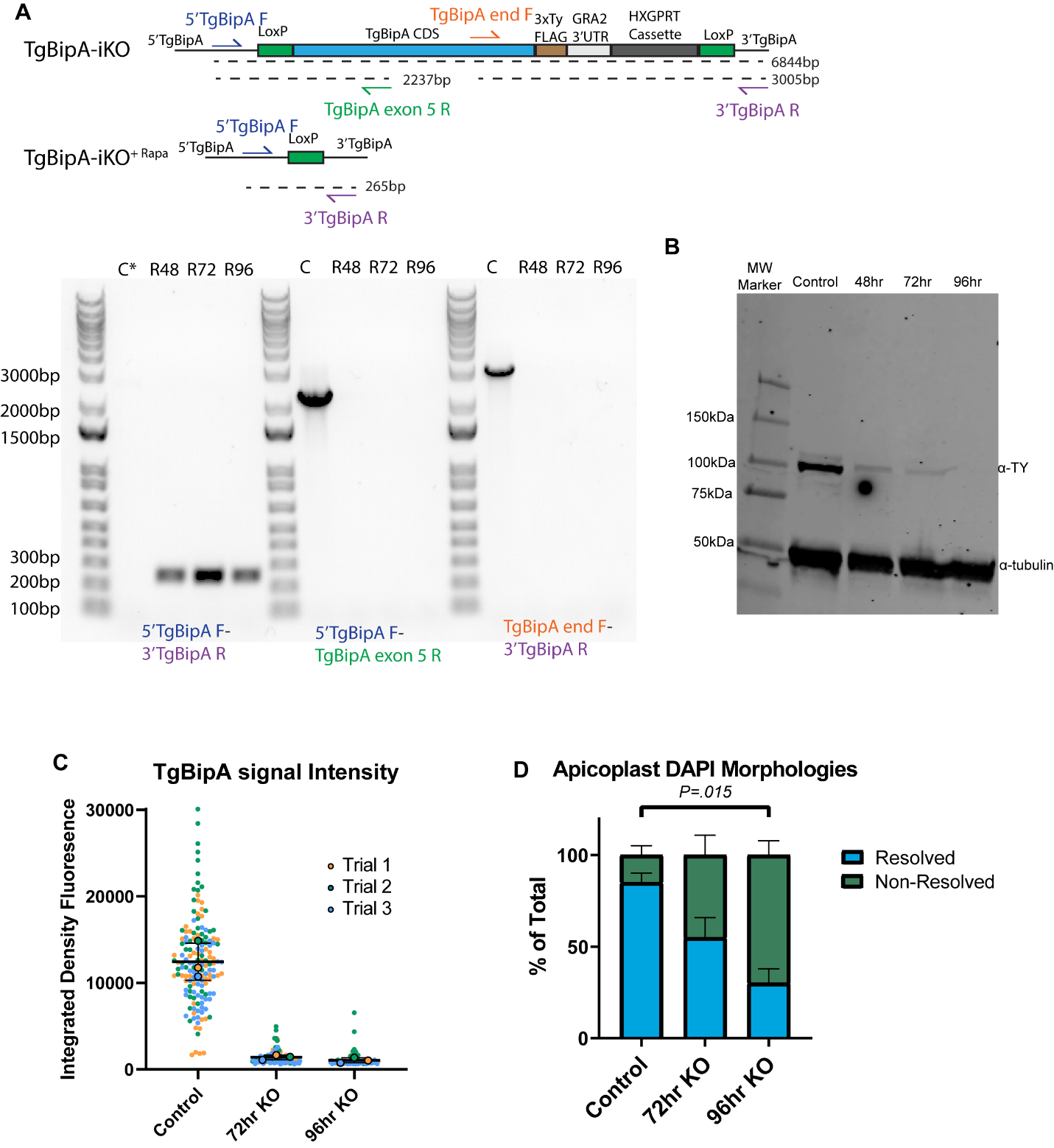


**Figure S3.**

A) *Upper*: Schematic of TgBipA-iKO and ΔTgBipA loci. Primer binding sites and PCR product sizes are indicated. *Lower*: Genomic PCR of TgBipA-iKO control (C) and ΔTgBipA 48, 72, or 96 hour post rapamycin treatment (R48, R72, R96). Predicted size of full loci is 6844bp and yielded no product (C* lane). B) Full western blot from Fig. 2A. C) TgBipA signal intensity in IF from experiment shown Fig. 2C, n of 50 cells from each of three independent experiments. D) % of parasites with resolvable apicoplast DAPI signal as determined IF for experiment shown in Fig. 3A and 3B.


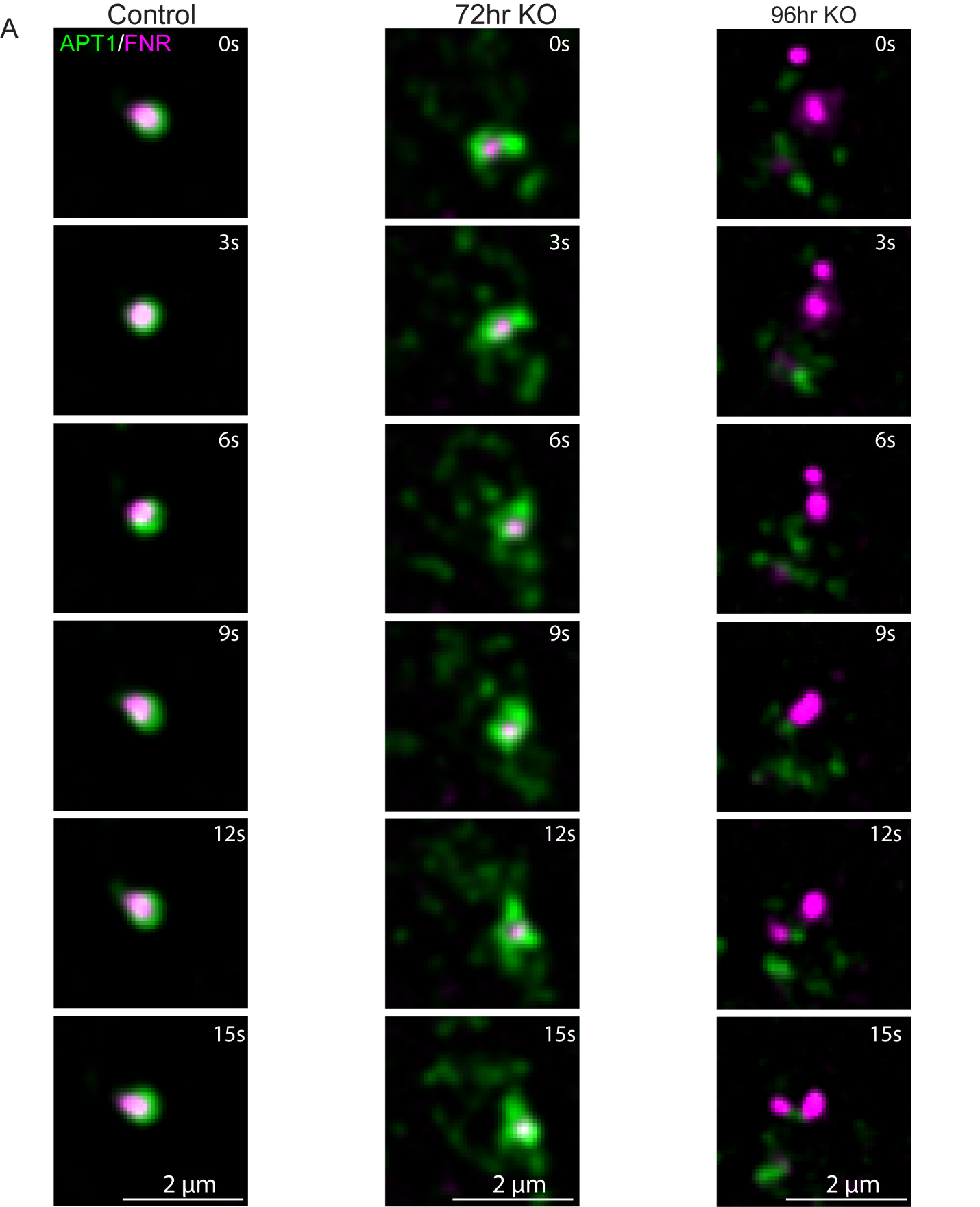


**Figure S4.**

Inset time lapse images from videos 1-3 (Control and TgBipA knockouts (72, and 96 hours after rapa treatment) expressing APT1-emfP and FNR-RFP.) Time in seconds is indicated in top right of image.

**
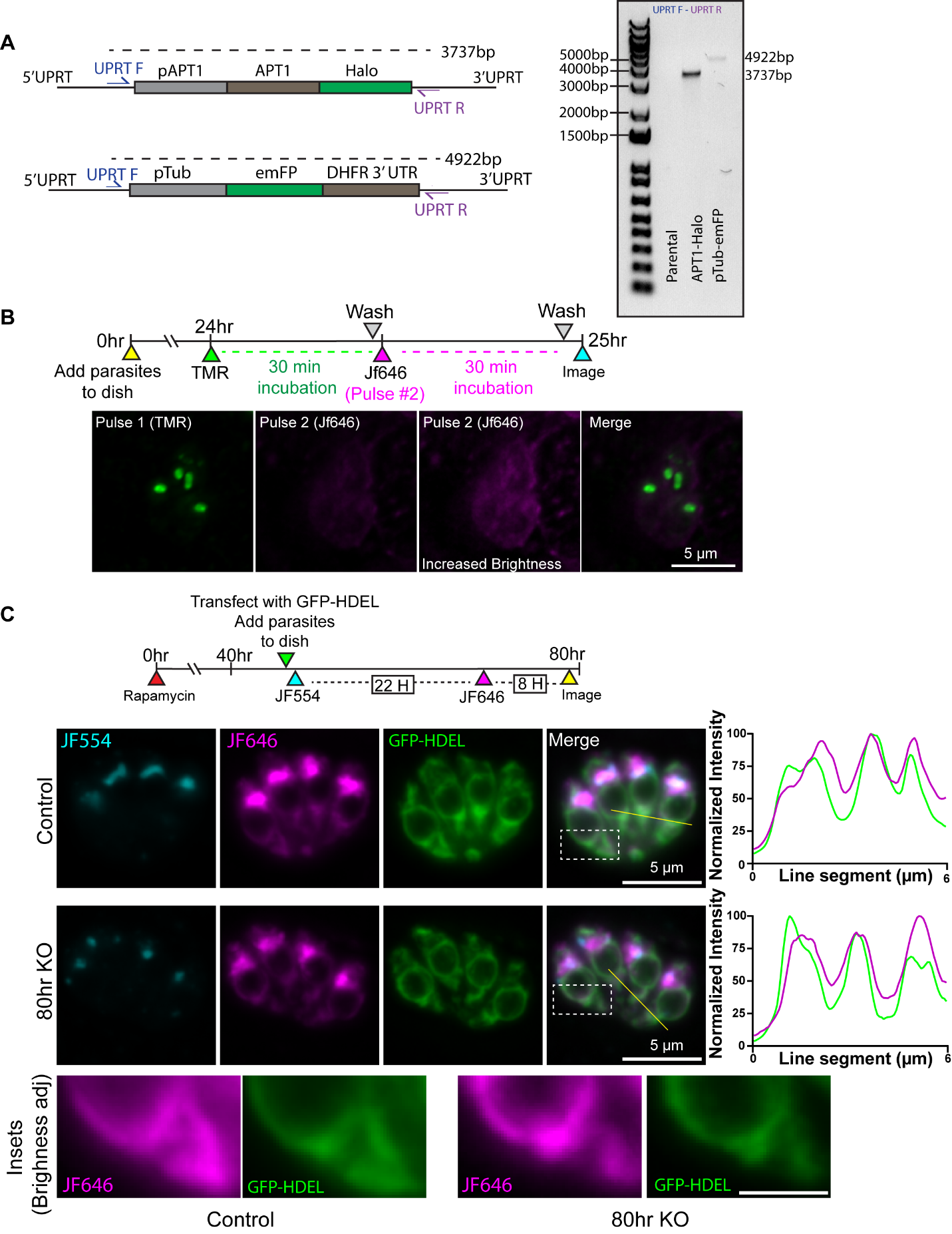
**

**Figure S5.**

A) *Left:* Schematic of the modified UPRT locus expressing APT1-Halo and emFP. *Right:* Genomic PCR confirmations of pAPT1-Halo and pTub-emFP inserted into the UPRT locus of TgBipA-iKO parasite line. B) Pulse chase assay of TgBipA-iKO::APT1-Halo parasites. Incubation with TMR dye labels Halo protein in the apicoplast. Immediate incubation and imaging with JF646 results in background levels of JF646 staining. Brightness was adjusted in the 2^nd^ JF646 panel to show faint Jf646 labeling in the ER. Scale bar = 5 µm. (C) *Upper:* Time course of the pulse chase assay. TgBipA-iKO::APT1-Halo parasites transiently expressing Sag1ΔGPI_GFP_HDEL as marker for the ER. Incubation with halo dyes indicated in timeline. *Lower:* Fluorescence microscopy images showing APT1 Halo labeled with JF554 (cyan) and JF646 (magenta), and GFP-HDEL (green). Inset panels show overlapping JF646 and GFP-HDEL signals. Brightness of JF646 panel was adjusted to highlight the dimmer ER localization. Dash white box indicates area used to make the inset. Yellow line indicates the area used to make the line scale, showing strong overlap between GFP and JF646 normalized fluorescent intensities.


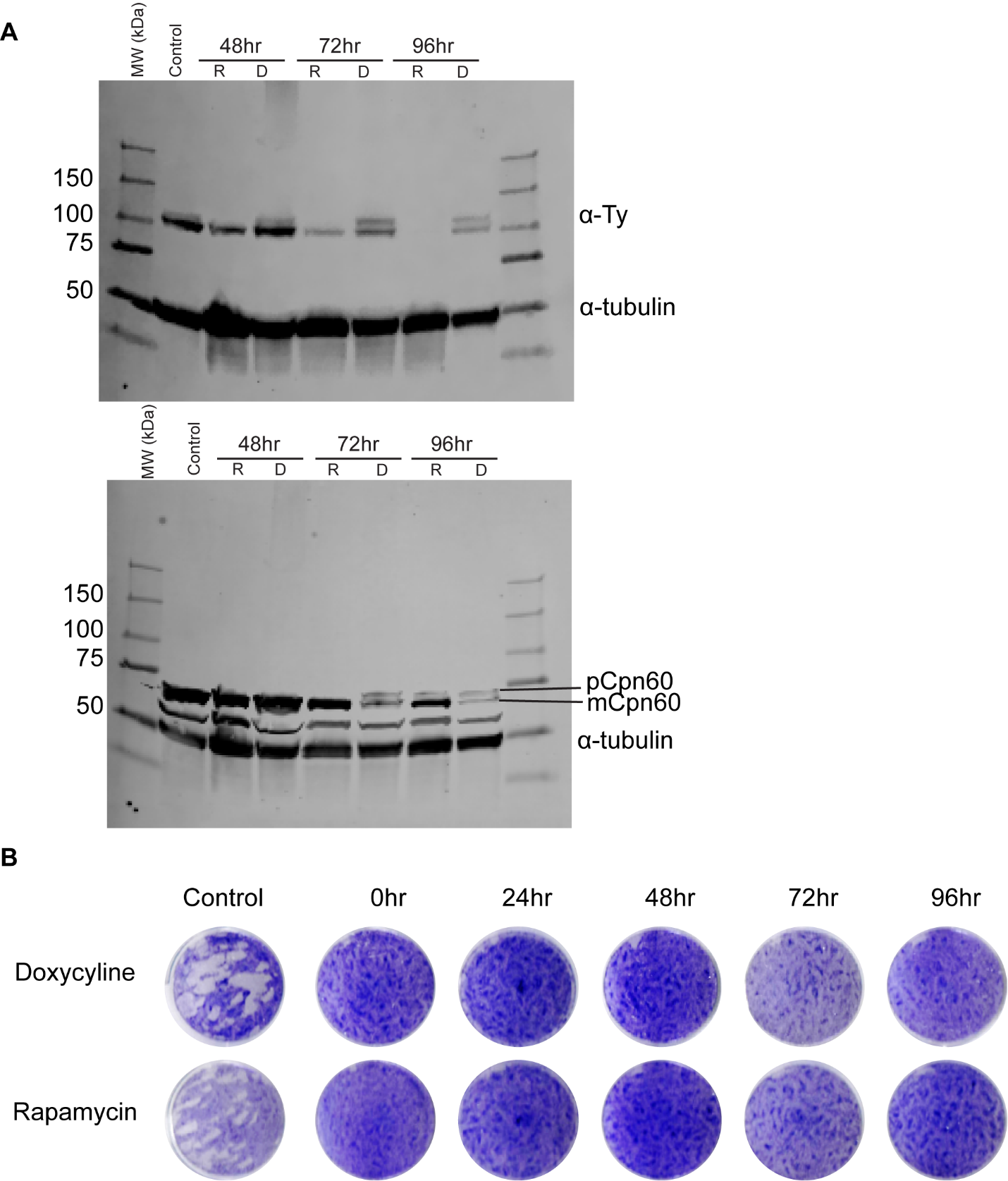


**Figure S6.**

A) Western blots of TgBipA-iKO parasites at 48, 72, and 96 hr post doxycycline (D) or rapamycin (R) treatment. Bands labeled for TgBipA (α-Ty), pre-mature Cpn60 (pCpn60) mature Cpn60 (mCpn60), and Tubulin (α-tubulin). B) Plaque assays of TgBipA-iKO parasites seeded at control, 0, 24, 48, 72, and 96 hr post doxycycline or rapamycin treatment.


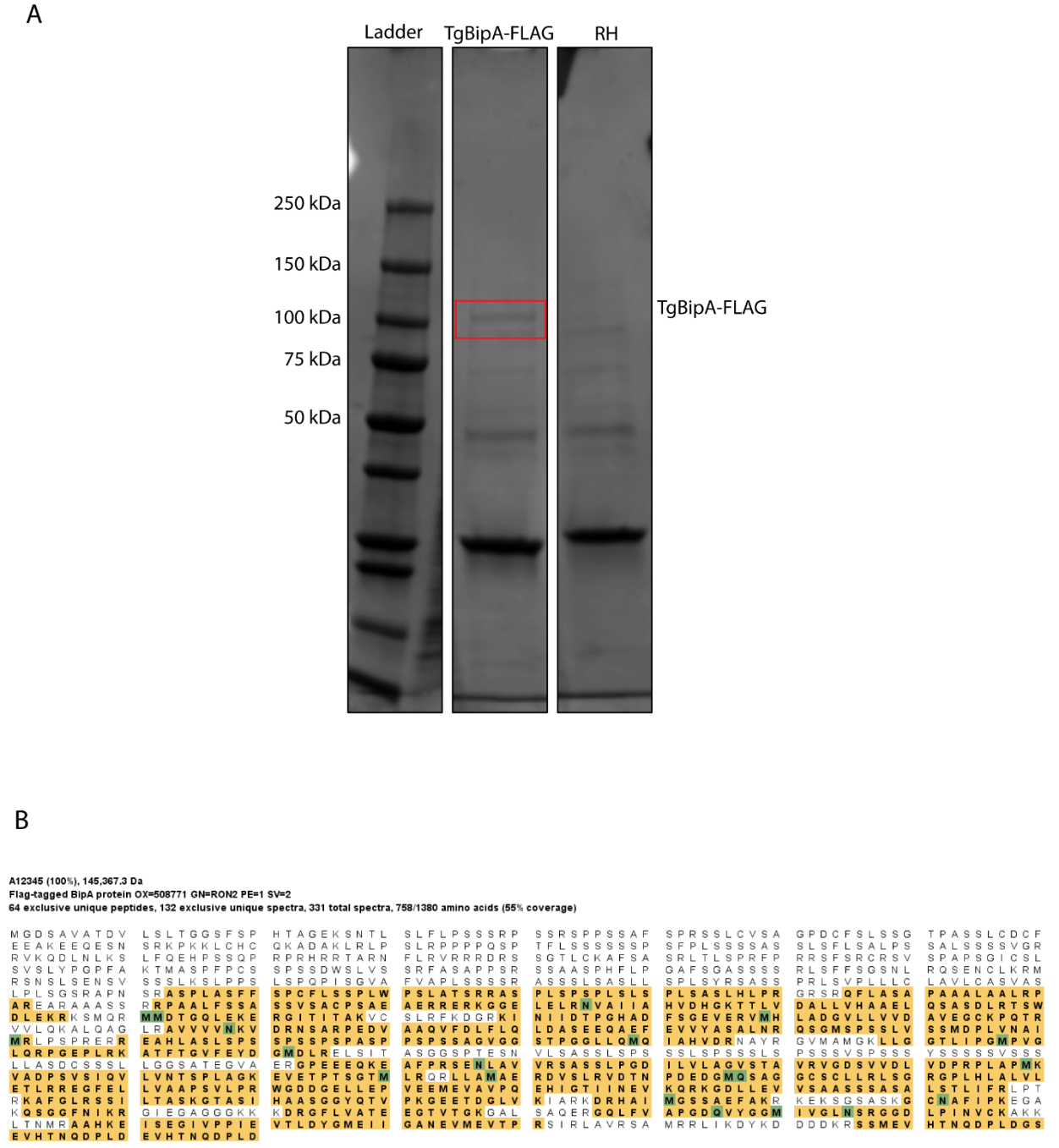


**Figure S7.**

A) Proteins purified by Flag immunoprecipitation from TgBipA-TyFLAG and RH control lysates were visualized on an SDS-PAGE gel stained with Coomassie. TgBipA-Flag is highlighted with a red box. B) TgBipA peptides identified by mass-spectrometry analysis. Identified residues highlighted in yellow, modified residues in green. No peptides were found in the N-terminus of the protein suggesting proteolytic processing.


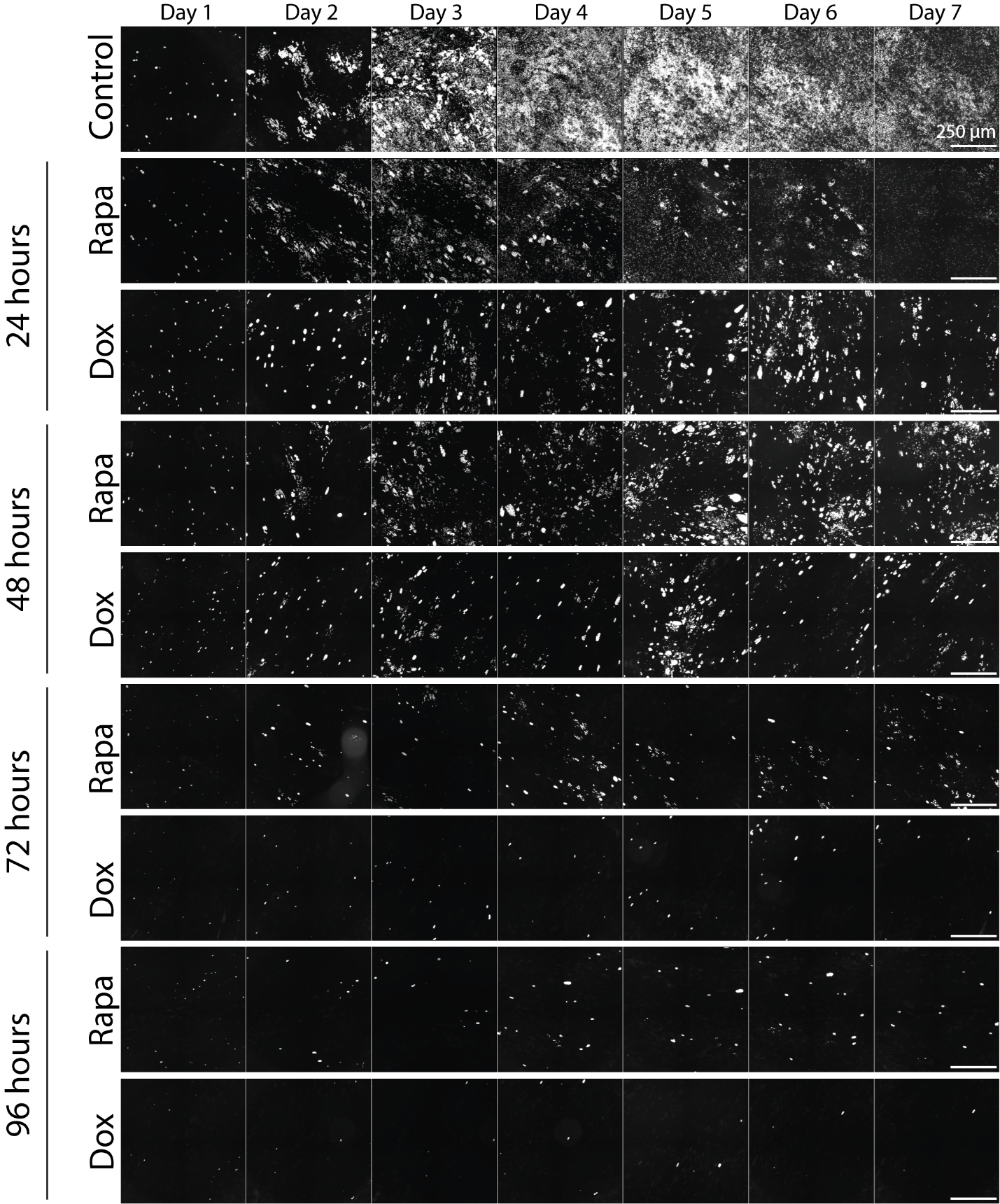


**Figure S8.**

Fluorescence microscopy images of taken every 24 hours during the growth assay shown in Figure 5C. Scale bar = 250 µm.


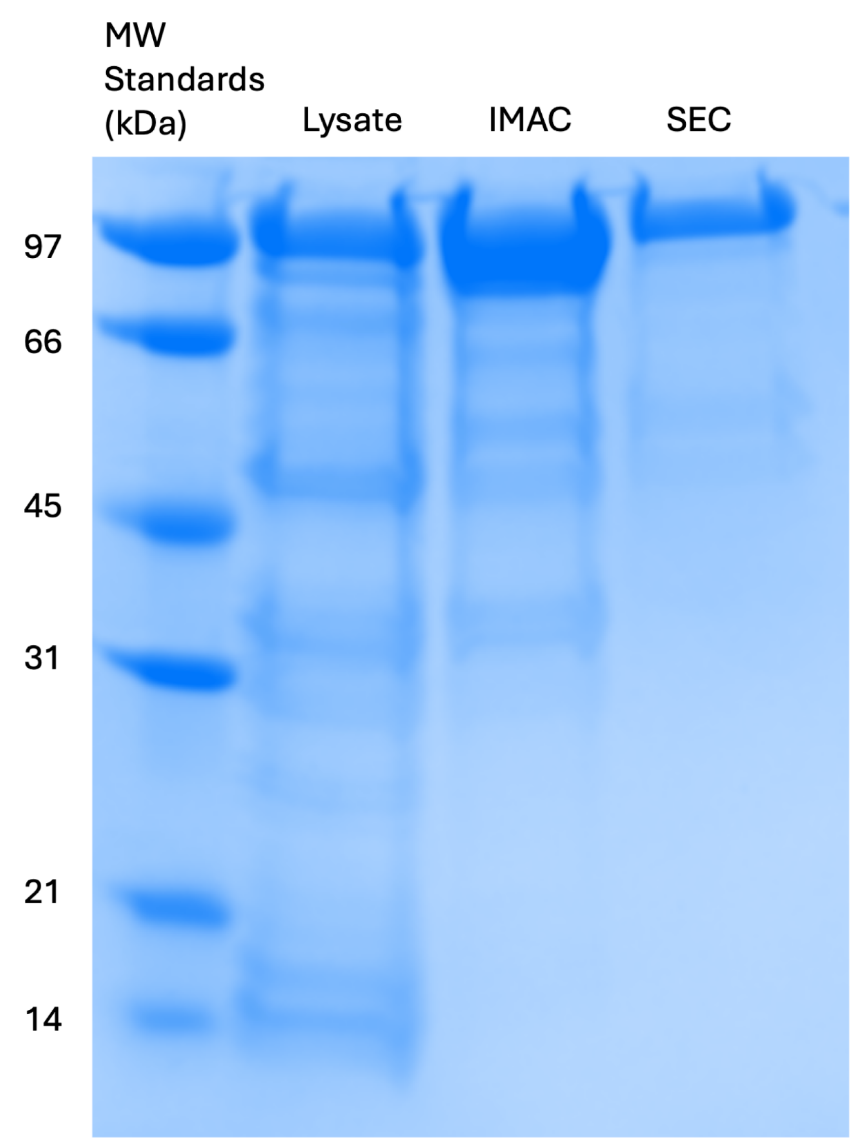


**Figure S9.**

Coomassie stained SDS-PAGE gel showing the purification of TgBipA (518-1333) expressed in bacteria. Lane 1, contains BioRad low-range SDS-PAGE molecular weight markers. Lane 2-Lysate: whole cell lysate of *E. coli* BL21 cells overexpressing His-tagged TgBipA(518-1333) solubilized in SDS-PAGE loading buffer and boiled for five minutes. Lane 3-IMAC: pooled fractions of the protein after eluted from a His-trap FF crude column (IMAC). Lane 4-SEC: pooled fractions from the size exclusion chromatography (SEC) that demonstrates protein purity of >95%.


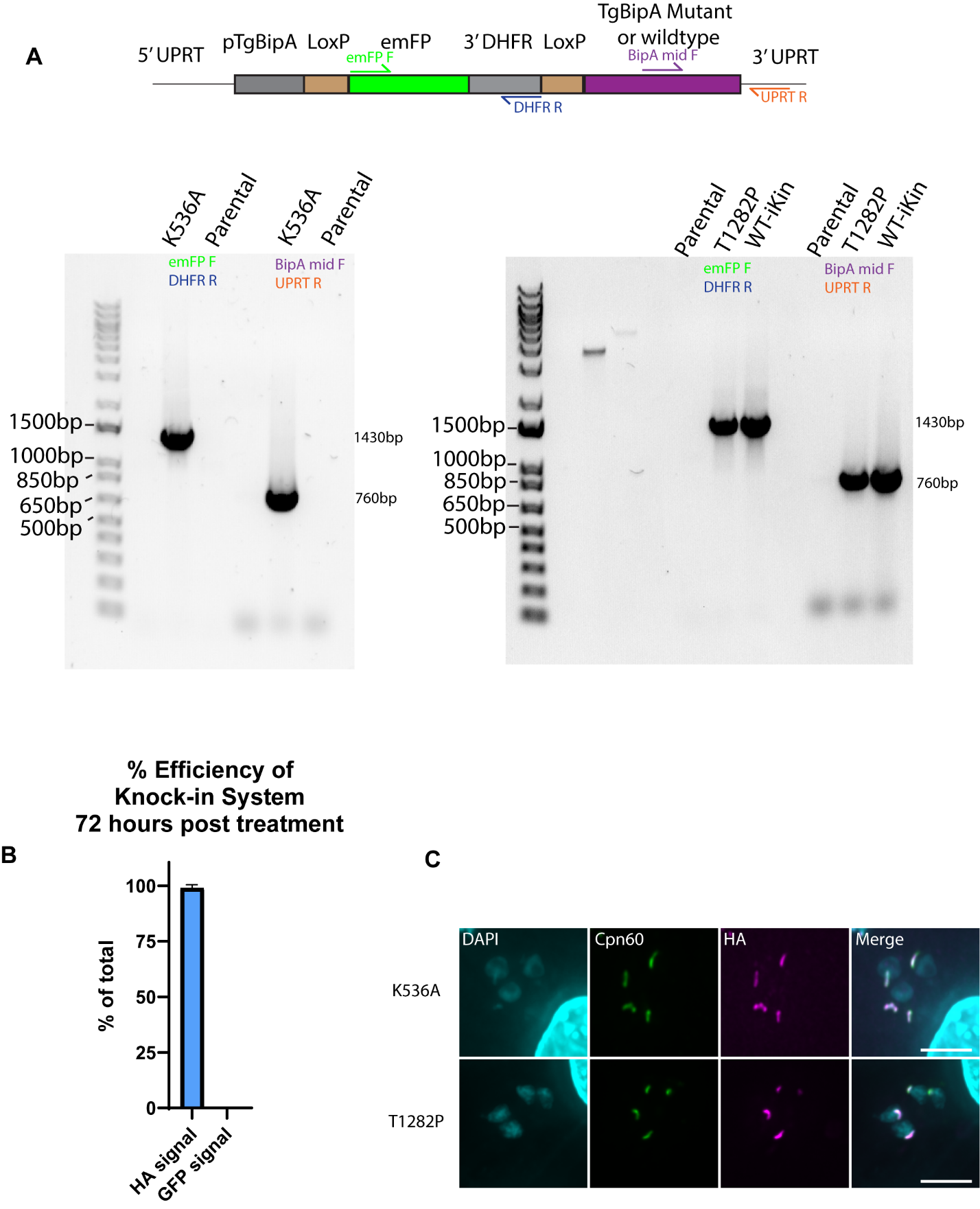


**Figure S10:**

A) *Upper:* Schematic of UPRT locus after integration of the knock-in system. *Lower:* Genomic PCR confirmations of TgBipA-iKin mutant rescue lines. B) % of parasites expressing HA-tagged construct which have lost GFP signal 72 hours after rapamycin treatment. n of 100 parasites in 2 independent experiments. C) Immunofluorescence images of K536A-ikin and T1282P-ikin parasites 24 hours after rapamycin treatment show mutations do not alter apicoplast localization. Scale bar = 5 µm.

**Video S1.**

Control TgBipA-iKO parasites expressing APT1-emFP and FNR-RFP imaged live for 30 seconds. Imaging speed 3 frames/sec. playback is 2x realtime. White box denotes inset region in Fig. S4. Scale bar = 5 µm.

**Video S2.**

ΔTgBipA (72H after rapa treatment) parasites expressing APT1-emFP and FNR-RFP imaged live for 30 seconds. Imaging speed 3 frames/second. Playback is 2x realtime. Brightness is adjusted to allow visualization of APT1 signal. White box denotes inset region in Fig. S4. Scale bar = 5 µm.

**Video S3.**

ΔTgBipA (96H after rapa treatment) parasites expressing APT1-emFP and FNR-RFP imaged live for 30 seconds. Imaging speed 3 frames/second. Playback is 2x realtime. Brightness is adjusted to allow visualization of faint APT1 and FNR signal. White box denotes inset region in Fig. S4. Scale bar = 5 µm.

**Table S1**. List of primers used in this study

**Table S2.** List of plasmids used in this study

**Table S3.** List of antibodies used in this study

**Table S4.** Mass-spectrometry data from anti-Flag pulldowns for TgBipA-FLAG and RH parasite lines. Combined data from 3 independent experiments.
